# Specialized signaling centers direct cell fate and spatial organization in a mesodermal organoid model

**DOI:** 10.1126/sciadv.ady7682

**Published:** 2025-11-28

**Authors:** Evangelia Skoufa, Jixing Zhong, Kelly Hu, Oliver Kahre, Georgios Tsissios, Louise Carrau, Antonio Herrera, Albert Dominguez Mantes, Marion Leleu, Alejandro Castilla-Ibeas, Hwanseok Jang, Martin Weigert, Gioele La Manno, Matthias Lutolf, Marian Ros, Can Aztekin

**Affiliations:** ^1^ School of Life Sciences, Swiss Federal Institute of Technology Lausanne (EPFL), Lausanne 1015, Switzerland.; ^2^ Friedrich Miescher Laboratory of the Max Planck Society, Tübingen 72076, Germany.; ^3^ Bioinformatics Competence Center, EPFL, Lausanne 1015, Switzerland.; ^4^ Department of Cellular and Molecular Signalling, Instituto de Biotecnología y Biomedicina de Cantabria (IBBTEC), CSIC-SODERCAN-University of Cantabria, Santander, Spain.; ^5^ Center for Scalable Data Analytics and Artificial Intelligence (ScaDS.AI), Technical University Dresden, Dresden, Germany.; ^6^ Institute of Human Biology (IHB), Roche Pharma Research and Early Development, Roche Innovation Center, Basel 4058, Switzerland.

## Abstract

Specialized signaling centers orchestrate robust development and regeneration. Limb morphogenesis, for instance, requires interactions between the mesoderm and the signaling center apical-ectodermal ridge (AER), whose properties and role in cell fate decisions have remained challenging to dissect. To tackle this, we developed mouse embryonic stem cell (mESC)–based heterogeneous cultures and a three-dimensional (3D) organoid model, termed budoids, comprising cells with AER, surface ectoderm, and mesoderm properties. mESCs were first induced into heterogeneous cultures that self-organized into domes in 2D. Aggregating these cultures formed mesodermal organoids with certain limb bud–like features in 3D, exhibiting chondrogenesis-based symmetry breaking and elongation. Using our organoids and quantitative in situ expression profiling, we uncovered that AER-like cells support nearby limb mesoderm and fibroblast identities while enhancing tissue polarization that permits distant cartilage formation. Together, our findings provide a powerful model to study epithelial signaling center-mesoderm interactions during morphogenesis and reveal the ability of signaling center AER cells to concurrently modulate cell fate and spatial organization.

## Teaser

Stem cell–derived mesodermal organoids reveal how signaling centers guide cell fate and tissue organization.

## INTRODUCTION

Specialized signaling centers are unique cell populations, defined by their diverse morphogen secretion capabilities, that transiently form and orchestrate morphogenesis during development and regeneration (*1*). A notable example is found in vertebrate limbs, where surface ectoderm cells differentiate into the signaling center apical-ectodermal ridge (AER) cells. The AER then establishes multiple morphogen gradients [including fibroblast growth factors (FGFs), bone morphogenic proteins (BMPs), wingless-related integration site proteins (WNTs), TGFBs (transforming growth factor–βs), and DELTAs (*2*, *3*)] simultaneously to influence the growth and differentiation of the underlying multipotent limb bud mesoderm. It has been postulated that mesodermal cells leaving the AER morphogen gradient zone differentiate into chondrocytes and fibroblasts (*2*). In parallel, signals from the non-AER surface ectoderm have been suggested to suppress chondrocyte differentiation (*4*). Although it is crucial to understand how these intricate cell-cell interactions are orchestrated to shape a developing limb, studies on signaling centers and limb morphogenesis are severely restricted by in vivo experimentation, which is constrained to tissue or gene-level examinations. The lack of viable cellular platforms poses a substantial challenge to using large-scale and quantitative measurements to study specialized signaling centers and their cell-cell interactions guiding limb development and regeneration.

Because of their well-controlled makeup, the ease of observation and perturbation and the scalability of organoids have become powerful models to yield numerous insights into the principles of morphogenesis (*5*). While many organoids and developmental phenomena rely on single-lineage self-organization (*6*), limbs require multilineage orchestration between the ectoderm and the mesoderm (*7*, *8*). However, simplified limb models have almost exclusively focused on the limb bud mesoderm (*9*–*13*), and the exploration of the ectoderm, including the AER, has been hampered by the unavailability of a suitable model (*14*). A recent three-dimensional (3D) limb bud model incorporated mesodermal and ectodermal cells, but it required manual dissection for organoid generation, fell short in emulating robust morphogenesis, and lacked comprehensive single-cell and functional characterization (*13*). Therefore, we developed and characterized a highly efficient in vitro mesodermal organoid model with certain limb bud–like features from mouse embryonic stem cells (mESCs) to dissect the self-organization propensities of distinct cell types and the specific roles of signaling center AER cells.

## RESULTS

### Stem cell–derived heterogeneous cultures resemble limb bud cell types

**Fig. 1.**
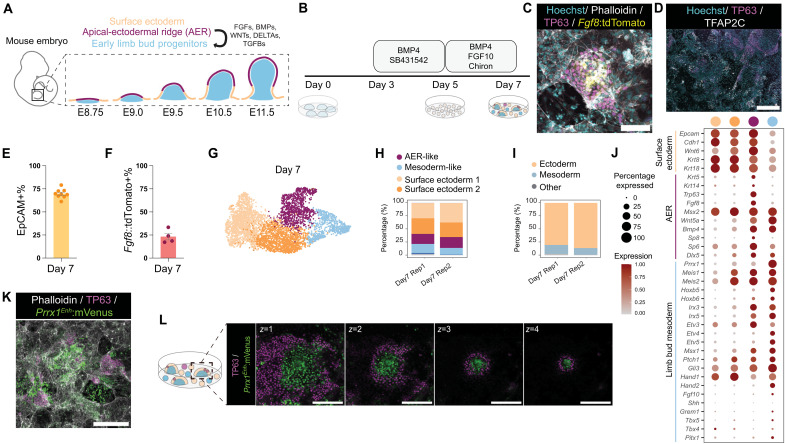
Induced heterogeneous cultures contain cells with signaling center AER, surface ectoderm, and mesoderm properties that self-organize into domes. (**A**) Schematic describing embryonic stages of mouse limb bud formation and the main cell populations involved in limb morphogenesis. (**B**) Schematic of the protocol generating cells with AER, surface ectoderm, and mesoderm properties from mESCs. (**C**) Representative confocal image of generated AER-like cells in day 7 cultures. Cyan: Hoechst; white: phalloidin; magenta: TP63; yellow: *Fgf8:tdTomato*. Scale bar, 100 μm. (**D**) Representative confocal image of day 7 heterogeneous cultures stained for immature surface ectoderm TFAP2C and basal epithelial stem cell marker TP63. Cyan: Hoechst; magenta: TP63; white: TFAP2C. Scale bar, 400 μm. (**E**) Bar plot showing flow cytometry quantification of epithelial cell marker EpCAM+ cells in day 7 cultures. Each dot represents one biological replicate. *N* = 9. (**F**) Bar plot showing flow cytometry quantification of *Fgf8:tdTomato*+ cells in day 7 cultures. Each dot represents one biological replicate. *N* = 4. (**G**) UMAP representation of scRNA-seq data of day 7 cultures displaying main cell clusters. See (H) color-coding. (**H** and **I**) Plots of the scRNA-seq cluster–based proportions of (H) generated cell types, with color-coding for AER-like, mesoderm-like, and surface ectoderm cells, and (I) lineages on day 7. For full information, see fig. S2E. Rep denotes replicate. (**J**) Dot plot showing surface ectoderm, AER, and limb bud mesoderm marker gene expression in the scRNA-seq clusters. Dot size represents the percentage of cells expressing each marker. (**K**) Representative confocal image of day 7 heterogeneous cultures generated from the *Prrx1^Enh^:mVenus* reporter mESC line. White: phalloidin; magenta: TP63; green: *Prrx1^Enh^:mVenus*. Scale bar, 500 μm. (**L**) Representative sequential *z*-stack confocal images of domes in day 7 cultures. Magenta: TP63; green: *Prrx1^Enh^:mVenus*. Images from left to right show the dome from bottom to top, denoted by *z* numbers. Scale bars, 200 μm.

We initially aimed to only generate signaling center AER cells from mESCs (Fig. 1, A and B). Adapting a published protocol (via SB431542 and BMP4) (*15*) to induce surface ectoderm–like cells—a precursor population to AER—yielded homogeneous epithelial cells (fig. S1, A to C). Subsequent activation of pathways required for AER induction, via BMP4 (*16*), FGF10 (*17*), and Wnt (*18*) agonist Chiron (hereafter, BFC) treatment, up-regulated well-established AER genes such as *Fgf8*, *Msx2*, and *Wnt5a* (fig. S1E) (*2*). Putative AER-like cells were detected as clusters on the basis of *Fgf8* and tumor protein P63 (TP63) staining on day 7 (fig. S1F). Generating and using an *Fgf8:tdTomato* reporter knockin line (fig. S1, G and H), we further found clusters of *Fgf8:tdTomato+*/TP63+ cells (Fig. 1C and fig. S1I). However, not all generated cells expressed these markers or the more immature surface ectoderm marker transcription factor AP-2γ (TFAP2C) (Fig. 1D). Furthermore, flow cytometry detected 69.7(±1.6)% epithelial cell marker EpCAM+ (epithelial cell adhesion molecule–positive) cells (Fig. 1E and fig. S1J) and 23.3(±3.6)% *Fgf8:tdTomato*+ cells (Fig. 1F and fig. S1K). The cultures also contained cells with fibroblast morphology (fig. S1D) and showed mesoderm-related *Prrx1* expression (fig. S1L), indicating the simultaneous formation of epithelial and mesenchymal populations.

### Single-cell characterization of heterogeneous cultures shows limb budlike identities

We reasoned that the generated AER-like cells were altering the local environment because of their ligand secretion capacity, resulting in the induction of heterogeneous cell types within the culture. To determine the cellular composition of our cultures, we performed time-course (0, 3, 5, and 7 days) single-cell RNA sequencing (scRNA-seq) and clustering-based cell type annotation (fig. S2, A to F). Days 0 to 3 of culture showed the expected transition from pluripotency states and the formation of neuroectodermal cells (fig. S2, A to F). Day 5 contained clusters expressing nonneural ectoderm (NNE) markers (e.g., *Krt8* and *Krt18*) (fig. S2, A to F), and some of these cells had an epithelial-to-mesenchymal transition signature (e.g., *Snai1/2* and *Zeb1/2*) (fig. S2, G and H), which might be associated with the induced mesodermal cells and fibroblasts following the BFC treatment.

Day 7 samples induced by the BFC treatment were mainly composed of cells with AER, surface ectoderm, or mesoderm features (Fig. 1, G to I, and fig. S2, A to F). The AER-like cluster was detected by associated transcription factors [e.g., *Sp6* and *Sp8* (*19*)] and various signaling ligands (e.g., *Fgf8*, *Bmp4*, and *Wnt5a*). Within our day 7 cultures, this cluster had *Trp63* expression as a unique marker (Fig. 1J and fig. S2D). Although some of these cells had high *Fgf8* expression (Fig. 1J), they largely lacked other AER-FGFs (*Fgf4*, *Fgf9*, and *Fgf17)* (fig. S3A), suggesting that they might represent an initial/early-forming AER state. Moreover, the generated AER-like cells were mainly in the G_1_ cell cycle phase (fig. S2F), consistent with the known mitotic inactivity of the AER (*20*, *21*). Compared to publicly available limb datasets from embryonic day 9.5 (E9.5) to E12.5 (*22*), these cells had the highest similarity to in vivo E9.5 AER (fig. S3B). Meanwhile, most BFC-induced cells showed surface ectoderm markers (e.g., *Krt8*, *Wnt3*, and *Wnt6*) (Fig. 1J and fig. S2D). None of these cells showed the expression of other relevant ectoderm or neural crest markers (fig. S3C).

Unexpectedly, the day 7 mesoderm cluster had lateral plate and early limb bud mesoderm marker expressions (e.g., *Hand1*, *Hand2*, and *Prrx1*) (Fig. 1J and fig. S2D) while lacking marker genes for other mesodermal populations (e.g., cranial mesoderm) (fig. S3E). Moreover, it did not have a high expression of *Shh* or *Grem1* nor hindlimb- or forelimb-associated genes (e.g., *Tbx4/5*) (Fig. 1J and fig. S2D). Unlike the in vivo highly proliferative limb bud mesoderm (*23*), it was mostly in the G_1_ phase (fig. S2F). To further examine their identity, we compared our data to reference E9.5-to-E12.5 mouse limb datasets (*22*). Correlation analysis showed putative matching across previously annotated (*22*) limb bud mesodermal populations, albeit the absolute values of correlations were low (fig. S3, B and D). To further test whether cultures contain limb bud mesoderm–like cells, we generated an mESC reporter line containing the *Prrx1* enhancer, traditionally used to label the limb bud mesoderm with high precision (*24*, *25*). Using this line, we identified that our protocol produces *Prrx1*^enh^+ fibroblast-like and more circular-looking cells (Fig. 1K and fig. S4, A and B). In summary, our protocol induced a heterogeneous population composed of cells that show notable similarities to AER, surface ectoderm, and to some degree, early lateral plate mesoderm and limb bud mesoderm identities.

### Heterogeneous cultures self-organize into 3D domes and budoids

Next, we examined the spatial organization of the generated AER-like and mesodermal cells in our 2D cultures. We found that the generated cells self-organized into 3D “domes” with TP63+ cells covering the outer layers of the domes and TP63+/*Fgf8*:*tdTomato*+ AER-like cells at the tip or outer bottom layers (Fig. 1L and fig. S4, C, E, and F). Moreover, the core of the domes was populated by *Prrx1^enh^*+ cells (Fig. 1L) or LEF1+ (lymphoid enhancer binding factor 1–positive)/TP63−/TFAP2C− cells with smaller nuclei (fig. S4, D to F), corresponding to the mesodermal cluster in the scRNA-seq dataset (fig. S4D). Together, these experiments suggested self-organization via AER-like and mesoderm interactions, generating domes in 2D cultures that extend into 3D and resemble limb buds.

**Fig. 2.**
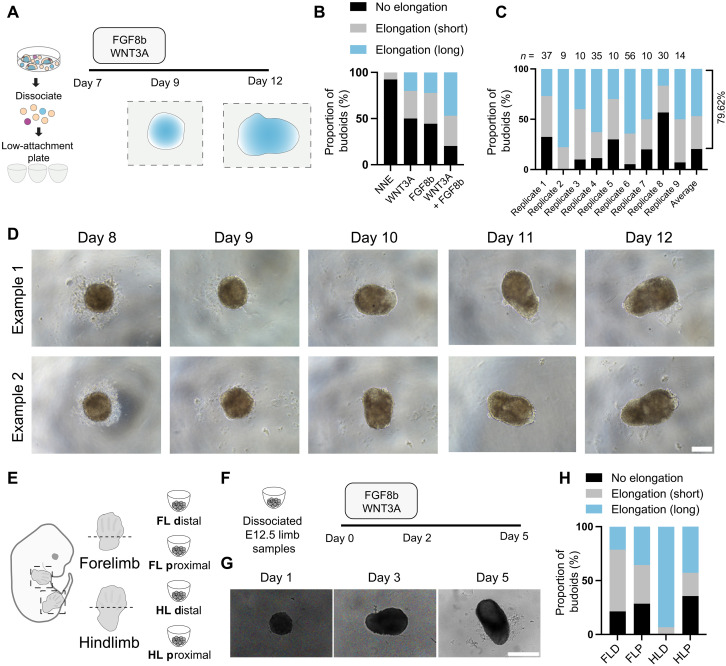
Stem cell–derived heterogeneous cultures and in vivo limb cells can form budoids. (**A**) Schematic of the budoid generation protocol. Treatments and morphological changes to cell aggregates at designated time points are outlined. (**B**) Bar graph displaying the proportion of budoids with no elongation, elongation (short), or elongation (long) after brief treatment with WNT3A (300 ng/ml), FGF8b (450 ng/ml), or a combination of both compared to control (NNE media). NNE: *n* = 39 budoids, *N* = 3; WNT3A (300 ng/ml): *n* = 10, *N* = 1; FGF8b (450 ng/ml): *n* = 9, *N* = 1; WNT3A (300 ng/ml) and FGF8b (450 ng/ml): *n* = 72, *N* = 2. (**C**) Bar graph showing the percentage of budoid structures achieving different elongation phenotypes under brief WNT3A (300 ng/ml) and FGF8b (450 ng/ml) treatment. The number above each set of bars (*n*) indicates the number of budoids analyzed within the replicate. The last column shows the weighted average. The total number of budoids analyzed was *n* = 211, *N* = 9 replicates. (**D**) Sequential bright-field images of examples of budoids from days 8 to 12 of the protocol. Scale bar, 100 μm. (**E**) Schematic representing dissection of forelimbs and hindlimbs into distal and proximal sections for budoid generation from primary cells. (**F**) Timeline schematic showing the dissociation of E12.5 limb samples and subsequent treatment with FGF8b and WNT3A at specified days to promote budoid formation and elongation.(**G**) Representative bright-field images of primary cell–derived hindlimb distal (HLD) budoids at days 1, 3, and 5 of the protocol. Scale bar, 200 μm. (**H**) Bar graph illustrating the proportion of bud-like structures from forelimb distal (FLD), forelimb proximal (FLP), hindlimb distal (HLD), and hindlimb proximal (HLP) regions, categorized by no elongation, elongation (short), or elongation (long) after 5 days of culture. Total number of budoids analyzed: FLD, *n* = 14, *N* = 3; FLP, *n* = 14, *N* = 3; HLD, *n* = 15, *N* = 3; HLP, *n* = 14, *N* = 3.

We subsequently asked whether our generated cells can also self-organize in 3D as free-floating cultures (Fig. 2A). Dissociating and seeding cultures in U-bottom low-attachment plates resulted in cells forming single spheric aggregates. Moreover, 5.08(±2.9)% of these aggregates displayed limited elongation and symmetry breaking, which is the transition from a symmetrical to asymmetrical state, here comprising the tissue-scale elongation of initially spherical homogeneous-looking aggregates but also referring to the development of polarized expression patterns within structures at the cellular level (Fig. 2B and fig. S5, A and B). Given that it is known that close-range ectoderm-mesoderm interactions are required for limb morphogenesis (*7*, *8*), we reasoned that the low degree of symmetry breaking might be due to insufficient ectodermal signals. A brief treatment of ectodermal signals FGF8b and WNT3A during aggregation increased the symmetry break efficiency to an average of 80.7(±5.9)% with a greater elongation phenotype (Fig. 2, B to D, and fig. S5, C to E). As our 3D structures were generated from cells with limb development characteristics and showed some morphological characteristics of limb buds, we termed them budoids.

Our results indicated that our stem cell–derived cells with certain limb developmental features can self-organize in 3D where they aggregate, subsequently break symmetry, and elongate. To further investigate the degree to which our cultures resembled in vivo limb bud cell types, we evaluated whether developing limb cells from embryonic mice could similarly self-organize in 3D in our setting. For this, we harvested and dissociated E12.5 mouse distal and proximal limbs (Fig. 2E) and exposed these to our protocol. All samples, particularly the hindlimb distal sample, underwent morphological organization similar to those seen in stem cell–derived budoids (Fig. 2, F to H, and fig. S6). Overall, these results demonstrated that our simplified setting can uncover the morphogenetic capacity of both stem cell– and in vivo–derived limb developmental cells.

### Budoids break symmetry through mesoderm-to-chondrocyte differentiation

**Fig. 3.**
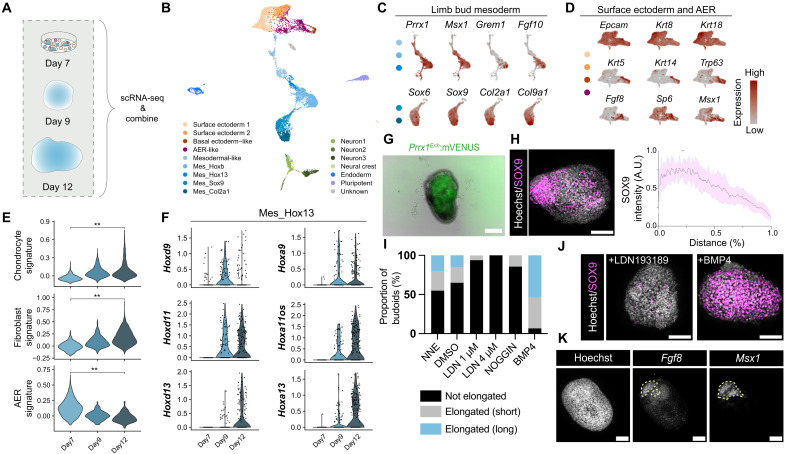
Budoids exhibit specific limb developmental hallmarks, and BMP-regulated mesoderm-to-chondrocyte differentiation mediates their symmetry breaking and elongation. (**A**) Schematic of the scRNA-seq analysis. (**B**) UMAP of the combined data illustrating diverse cell populations identified by scRNA-seq. (**C**) Feature plots displaying the limb bud mesoderm and chondrogenesis gene expression in mesodermal clusters indicated by colored dots. (**D**) Feature plots showing surface ectoderm and AER gene expression in ectodermal clusters indicated by colored dots. (**E**) Violin plots depicting time-course gene set enrichment scores for chondrocyte- and fibroblast-associated signatures in combined mesodermal clusters and AER-associated signatures in combined ectodermal clusters. Day 7 and 12 data were compared via two-sided Wilcox tests. **P < 0.01. (**F**) Violin plots demonstrating time-course 5′ *HoxA and HoxD* gene activation in the Mes_Hox13 cluster. Each dot represents one cell. (**G**) Representative bright-field– and fluorescence-overlaid image of a day 12 *Prrx1^Enh^:mVenus* line budoid. Green: *Prrx1^Enh^:mVenus*. Scale bar, 100 μm. (**H**) (Left) Representative max-projection confocal image displaying SOX9 polarization within a day 13 budoid. Gray: Hoechst; magenta: SOX9. Scale bar, 100 μm. (Right) Normalized SOX9 intensity across the major axis of day 12 budoids (*n* = 10, *N* = 4). A.U., arbitrary units. (**I**) Bar chart displaying the weighted average of day 13 budoid proportions exhibiting no elongation, elongation (short), or elongation (long) after treatment with NNE (no treatment control; *n* = 20, *N* = 2), DMSO control (*n* = 20, *N* = 2), 1 or 4 μM LDN193189 (LDN 1 μM: *n* = 16, *N* = 2; LDN 4 μM: *n* = 19, *N* = 2), recombinant NOGGIN (300 ng/ml) (*n* = 14, *N* = 2), or recombinant BMP4 (300 ng/ml) (*n* = 35, *N* = 2). (**J**) Max-projection confocal images of day 13 budoids treated with LDN 1 μM or BMP4 (300 ng/ml). Gray: Hoechst; magenta: SOX9. Scale bars, 100 μm. (**K**) Confocal image of a budoid showing polarized *Fgf8* mRNA– and *Msx1* mRNA–positive cells. Please note this phenotype is seen in 3 of 10 budoids. Scale bars, 100 μm.

To unravel cell fate changes during stem cell–derived budoid morphogenesis, we performed scRNA-seq and clustering analysis on pooled budoid samples before (day 9) and after symmetry breaking (day 12) and combined the generated datasets with our 2D heterogeneous culture data as a starting point (Fig. 3, A and B, and fig. S7). scRNA-seq demonstrated the enrichment of mesodermal cells (Fig. 3, C to G, and fig. S7B), with budoids containing cells with multipotent limb bud mesoderm markers (e.g., *Msx1*, *Grem1*, and *Fgf10*) (Fig. 3, C and E to G, and fig. S7F) (*26*) including sequential activation of *Hox* clusters (Fig. 3F). Budoids contained cells displaying broad transcriptome-wide similarity to in vivo limb bud mesodermal cells, although individual clusters most closely resembled different developmental stages in published in vivo scRNA-seq datasets (fig. S8, A and B). Moreover, when we projected budoid-derived cells onto an in vivo limb bud reference (*22*), ectodermal cells, including AER cells, and mesodermal cells appeared with their in vivo counterparts, except some mesodermal cells aligned with ectodermal clusters (fig. S8C), consistent with our pair-wise comparison (fig. S8, A and B).

We then showed that budoids mainly comprise *Prrx1^Enh^*+ cells (Fig. 3G), in agreement with our single-cell analyses. Anterior-posterior patterning–related *Shh* was seen in a few cells (fig. S7F). Mesodermal cells were agnostic for hindlimb (*Tbx4* and *Pitx1*) or forelimb gene profiles (*Tbx5*) (fig. S7F), with no single cell showing abundant *Tbx4* and *Tbx5* expression simultaneously (figs. S7F and S8D). Consistent with the hypothesis that budoids contain multipotent limb bud mesoderm–like cells, we found budoids to express chondrocyte-associated (*Sox9* and *Col2a1*) and fibroblast-associated genes (*Col1a1*, *Col3a1*, and *Dpt*), including tenocytes (*Scx* and *Lum*) and pericytes (*Rgs5*, *Acta2*, and *Des*) (fig. S7B).

Critically, post–symmetry breaking, scRNA-seq indicated an increase in chondrocyte populations in budoids (fig. S7B). This enrichment was corroborated by our imaging results where we observed the emergence of a polarized SOX9+ (SRY-box transcription factor 9–positive) domain (Fig. 3H), which was also observed in in vivo–derived budoids (fig. S8E). Thus, we hypothesized that budoid symmetry breaking is mediated by chondrogenesis. Suppression of the well-established chondrogenesis regulator BMP pathway by the addition of recombinant NOGGIN or pharmacological inhibition via LDN193189 impaired symmetry breaking and elongation (Fig. 3I), and these budoids had fewer SOX9+ cells (Fig. 3J). In contrast, the addition of recombinant BMP4 showed the opposite effect (Fig. 3I), with almost all organoids elongated and entirely populated by SOX9+ cells (Fig. 3J). Collectively, these results demonstrated that the budoid symmetry break is mediated by limb mesoderm–to–chondrocyte differentiation, which is regulated by BMP pathway activity.

Substantial ectodermal reduction was evident during budoid formation (fig. S7B), reinforced by the observation that budoids had limited E-CADHERIN+ and TP63+ cells (fig. S8F). We also detected the same phenotype with in vivo–derived budoids (fig. S8E), suggesting that our culture conditions will require further optimization for ectodermal cell aggregation and survival. Moreover, the detected ectodermal cells had a lower AER signature compared to day 7 samples (Fig. 3E). Nonetheless, a spatially distinct AER marker *Fgf8* was identified as coexisting with regions of *Msx1* expression as has been seen in vivo (*27*), albeit this phenotype was only observed in some budoids (Fig. 3K). Budoids also had some nonlimb lineages (e.g., *Sox17*+ endodermal and *Nanog*+ stem cells) (fig. S7B), presumably due to differentiation artifacts that are commonly seen in stem cell–derived organoids (*28*). Collectively, these findings suggest that budoids are organoids, mainly composed of mesodermal cells, that show some characteristics of limb developmental processes.

### Mesodermal cells show morphogenic properties for main budoid morphology

**Fig. 4.**
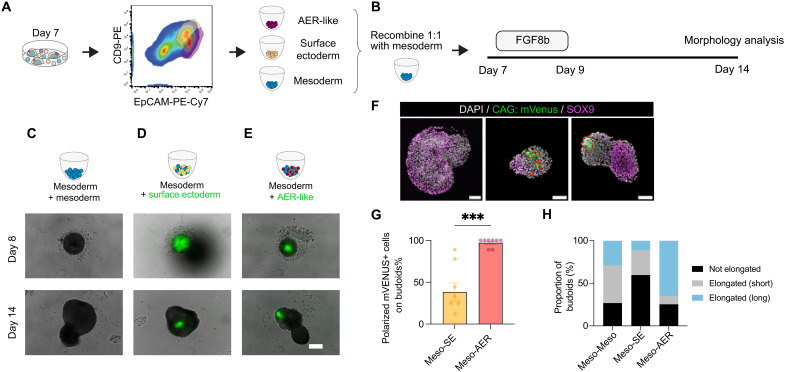
Recombinant budoids uncover differential roles of AER-like and surface ectoderm cells in mesoderm symmetry breaking. (**A**) Schematic showing an example of a FACS strategy to enrich specific cell types. (**B**) The experimental design for recombinant budoids is outlined and followed by assays for morphological changes and spatial sequencing. (**C** to **E**) Overlaid bright-field and fluorescence images taken on days 8 and 14 show (C) mesoderm recombined with mesoderm cells, (D) mesoderm recombined with surface ectoderm–like cells, and (E) mesoderm recombined with AER-like cells. Please note that the surface ectoderm–like and AER-like cells are derived from the *CAG:mVenus* line, serving as a fluorescent marker for tracking. Nonaggregated and shed cells, especially in ectodermal cells, are seen on day 8 samples. Distinct polarized *mVenus*+ AER-like cells are seen on day 14 in (E). Scale bar, 100 μm. (**F**) Representative confocal images of sectioned recombinant budoids. mVENUS+ ectodermal cells are circled with a red dashed line. Please note distinct polarized mVENUS+ AER-like cells. Magenta: SOX9; green: *CAG:mVenus*; Hoechst: DAPI. Scale bars, 250 μm. (**G**) Bar plot showing a polarized *mVenus* group of cells on recombinant budoids. Each dot represents the average phenotype per replicate. Total number of budoids analyzed: Meso-SE, *n* = 60, *N* = 8; Meso-AER, *n* = 63, *N* = 8. ***P < 0.001. (**H**) Bar chart with a weighted average of the proportion of recombinant budoids for their elongation. Total number of budoids analyzed: Meso-Meso, *n* = 80; Meso-SE, *n* = 78; Meso-AER, *n* = 63, from *N* = 8.

Next, we aimed to leverage budoids as a simplified system to dissect the effect of signaling center AER-like cells. For this, we first established a cell sorting strategy (Fig. 4A and fig. S9), informed by the scRNA-seq dataset (fig. S9A), to enrich mesodermal (EpCAM^Neg^), surface ectoderm–like (EpCAM^High^/CD9^High^), or AER-like (EpCAM^High^/CD9^Low^) cells. Enriched AER-like and mesoderm populations expressed the markers *Fgf8* and *Prrx1*, respectively (fig. S9D), and they were devoid of the pluripotent stem cell marker *Nanog*, while ectoderm-enriched populations displayed some expression of endoderm-associated *Sox17* (fig. S9E). These results established a successful enrichment strategy with minimal contaminant populations for cell type–specific assessments.

We then tested the generation of budoids from only mesoderm, AER-like, or surface ectoderm–like cells and found that they all had disintegration and aggregation problems (fig. S10A). However, generating aggregates with a brief FGF8b exposure revealed that mesoderm-enriched cells alone can break symmetry and elongate (Fig. 4C and fig. S10, B and C), although they showed variability in size across experimental sets (fig. S10B). In contrast, the AER-like enriched population did not properly aggregate (fig. S10, A and B), albeit there was a minor improvement with brief FGF8b treatment (fig. S10C). These phenotypes aligned with AER-like cells tested by sorting and aggregating day 7 *Fgf8:tdTomato*+ cells (figs. S9F and S10D). Surface ectoderm–like enriched cells were able to aggregate more with a brief FGF8b treatment but did not show symmetry breaking or elongation (fig. S10C). In summary, we concluded that mesoderm-enriched cells can display morphogenesis by breaking symmetry and elongation upon brief exposure to FGF8b, whereas ectodermal cells alone do not have a similar morphogenic capacity.

### AER-like cells affect budoid morphology and spatial organization

Then, we devised “recombinant budoids” to uncover the impact of signaling center AER-like cells on the mesoderm-enriched population (Fig. 4B). Recombinants were generated by combining equal numbers of fluorescence-activated cell sorting (FACS)–sorted mesodermal and AER-like cells. Similarly, we recombined the mesoderm with surface ectoderm–like or mesodermal cells as controls. Moreover, we generated AER-like and surface ectoderm–like cells from a constitutively active *mVenus*-expressing line to trace them. Because of aggregation problems (fig. S10, A to E), all conditions received FGF8b treatment for 2 days and were grown for an additional 5 days before being examined for morphological features (Fig. 4B).

Although we seeded the same number of cells for recombination, we observed different-sized aggregates at 1 day postseeding under all conditions (Fig. 4, C to E, and fig. S11A), possibly because of cell type–specific aggregation capacities (fig. S10, A and B, and fig. S11A). The mesoderm with AER-like recombination had a smaller area and contained fewer mVENUS+ cells at day 1 compared to other recombinants (Fig. 4, C to E, and figs. S10F and S11A). Recombined AER–like or surface ectoderm–like cells did not expand and were further eliminated from the structures over time (Fig. 4, D and E, and fig. S10F), consistent with the reduction of ectodermal cells in previous budoid experiments (figs. 7B and S8, E and F). Moreover, these ectodermal cells were typically seen together and engulfed in the budoids (Fig. 4F), probably due to cell sorting and differential adhesion properties favoring ectoderm internalization, as suggested before (*29*). A total of 97.2(±1.8)% of the analyzed recombinant budoids had polarized mVENUS+ AER-like cells, while the same phenotype was only seen in 38.4(±10.9)% of the mesoderm with surface ectoderm–like recombinations (Fig. 4, D, E, and G, and fig. S10F). We then evaluated the fold change in area from initial aggregates to final structures as a proxy for their growth. All conditions showed comparable values (fig. S11A), indicating that the AER-like recombination in this setup does not noticeably affect the growth rate.

The mesoderm with AER-like recombinant budoids had substantial symmetry breaking and elongation compared to the mesoderm with surface ectoderm recombinants (Fig. 4H and fig. S11B), which remained in a more circular morphology (Fig. 4D and fig. S10F). Given our results suggesting that chondrogenesis is associated with symmetry breaking (Fig. 3I and fig. S7B), the inability of surface ectoderm–like recombination to break symmetry and elongate may be consistent with the previously proposed function of ectodermal cells in counteracting chondrogenesis (*4*, *30*). However, we did not observe a similar phenotype with the AER-like recombination (Fig. 4H and fig. S11B). To determine whether this was due to the polarized spatial segregation of AER-like cells (Fig. 4E and fig. S10F) and their potential local impact, we evaluated the chondrogenesis marker SOX9 (*31*) and the fibroblast/limb bud mesoderm marker TWIST1 (*32*). These markers mostly showed a mutually exclusive expression pattern across all samples, constituting most of the cells in structures (fig. S11C). Abundant SOX9+ cells were seen in mesoderm-enriched samples (Fig. 4F and fig. S11C). Notably, AER-like and SOX9+ cells were mostly polarized at opposite ends of the structures (Fig. 4F and fig. S11C), suggesting local suppression while permitting this differentiation in a distance. In contrast, mVENUS+ surface ectoderm–like cells could be found with some SOX9+ cells in close proximity without clear polarization (Fig. 4F and fig. S11C). Thus, these recombination experiments confirmed the functional properties of ectodermal cells, consistent with previous suggestions that the ectoderm impairs chondrogenesis (*4*, *30*). Moreover, our experiments also distinguished specific effects of AER from the surface ectoderm: AER-like cells impaired chondrogenesis only for neighboring cells without disrupting the mesodermal morphogenetic capacity, whereas surface ectoderm recombination directly reduced chondrogenesis-based symmetry breaking. Overall, our recombinant budoid strategy allowed us to reveal cell-cell interactions that are difficult to dissect in vivo and how the ectoderm influences the spatial segregation of cell fate decisions and tissue organization.

### AER-like cells promote tissue polarization in recombinant budoids

**Fig. 5.**
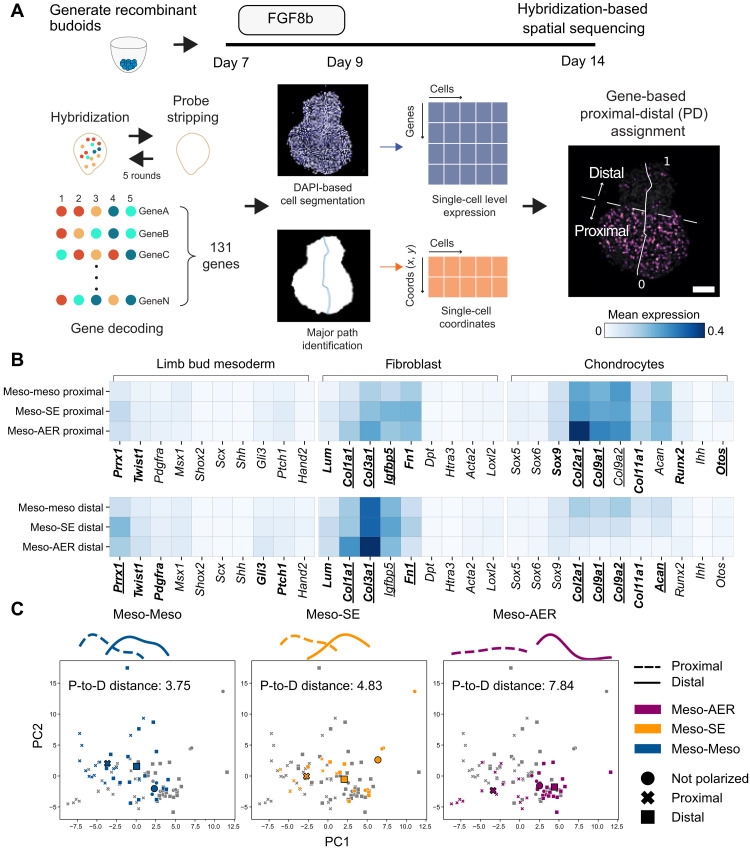
In situ gene expression profiling shows that AER-like cells promote spatially distinct polarized fibroblast and cartilage domains. (**A**) Schematics describing the HybISS methodology and subsequent analysis, leading to the unbiased identification of proximal and distal domains of day 14 recombinant budoids. Please see Materials and Methods for details. The major axis is labeled with a line. A value from 1 to 0 represents distal to proximal directions. The dashed line splits the recombinants into distal and proximal domains. Scale bar, 100 μm. (**B**) The heatmap displays average expression levels of genes associated with limb bud mesoderm, fibroblasts, and chondrocytes in the recombinant budoid single cells within proximal and distal domains. Genes with statistically significant differences are highlighted, with bold indicating a significant difference between mesoderm-AER and mesoderm-mesoderm comparisons, while underlining signifies significant differences between mesoderm-AER and mesoderm-surface ectoderm. Number of HybISS sections analyzed: Meso-Meso, *n* = 20, *N* = 4; Meso-SE, *n* = 12, *N* = 3; Meso-AER, *n* = 20, *N* = 4. (**C**) PCA of sectioned recombinant budoids illustrates the separation between the proximal (P) and distal (D) domains. Proximal domains of individual recombinant sections are denoted by **×**, and distal domains are denoted by ■. Sections without polarization are denoted by **•**. Density plots showing the distribution of samples with respect to PC1 are shown at the top of the PCA plots. The distributions of distal and proximal samples are represented with a straight line and dashed line, respectively. The distance between the centroids of the distal and proximal samples (black-framed bold symbols) was quantified and displayed. Samples are color-coded. Number of HybISS sections analyzed: Meso-Meso, *n* = 20, *N* = 4; Meso-SE, *n* = 12, *N* = 3; Meso-AER, *n* = 20, *N* = 4.

We then investigated the molecular characteristics of our recombined structures using quantitative hybridization-based in situ sequencing (HybISS) (*33*) assaying 131 limb development–related cell type and signaling pathway genes (Fig. 5A and fig. S12). Leveraging the single-cell resolution of HybISS alongside spatial cellular coordinates, we separated individual samples into two domains (Fig. 5A). Across all recombinants, we defined one side of the structures showing enrichment for chondrogenic markers (e.g., *Sox9*, *Col2a1*, *Col9a1*, and *Col9a2*) (Fig. 5C and fig. S13A) as the proximal domain. Meanwhile, the opposite side, the distal domain, showed a higher expression of limb bud mesoderm, fibroblast (e.g., *Prrx1*, *Msx1*, *Twist1*, *Lum*, *Col1a1*, and *Col3a1*) (Fig. 5B and fig. S13A), and signaling genes (e.g., *Bmp4*, *Bmp7*, *Id2*, *Id3*, *Wnt5a*, *Wnt6*, *Rspo2*, *Rspo3*, *Axin2*, *Tcf7*, *Fgf7*, *Dusp1*, *Spry2*, and *Spry4*) (fig. S13, B to F). Therefore, recombinant budoids have two distinct domains that resemble spatial features of limb buds: the proximal domain enriched for cartilage identity and the distal domain with limb bud mesoderm and fibroblast states and high signaling activity.

Focusing on ectodermal cells, we found a few distally enriched potential remnants of AER-like (*Krt5*+) cells, while surface ectodermal–like (*Krt8+*/*Krt18+*) cells showed no clear enrichment for a specific location (fig. S14A). Previously reported AER-induced genes such as *Msx1*, *Bmp4*, *Wnt5a*, *Gli3*, and *Axin2* (*27*) were also enriched at the distal domains of AER recombinants (fig. S14B), further confirming the functionality of generated cells and their lasting effect. AER-like cells with mesoderm recombinants showed high expression for fibroblast (*Prrx1*, *Twist1*, *Lum*, *Col3a1*, *Igfbp5*, and *Col1a1*) and limb bud mesoderm genes (*Fgf10*, *Msx1*, and *Gli3*) across both domains with distal enrichment (Fig. 5B and fig. S13A). In contrast, these signatures were either reduced or at comparable levels in the surface ectoderm with mesoderm recombinants and even more significantly reduced in mesoderm-only recombinants (Fig. 5B and fig. S13A). Instead, mesoderm-only recombinants had high chondrogenesis gene expression (e.g., *Col2a1*, *Acan*, *Col9a1*, and *Col9a2*) in both domains (Fig. 5B and fig. S13A), further supporting the notion that AER-like cells in budoids influence the patterned expression of chondrogenesis genes.

Last, performing principal components analysis (PCA) on samples classified into proximal and distal domains identified that the AER-like cells increased polarization between domains, while the other conditions showed more mixing (Fig. 5C). Together, these results uncovered that signaling center AER-like cells promote or sustain the nearby limb bud mesoderm and fibroblast identities, while suppressing nearby chondrogenesis, and affect spatially distant cartilage formation by promoting polarization.

## DISCUSSION

Here, we present a highly scalable and novel deconstructed approach to study signaling center-mesoderm interactions. The budoid methodology provides a unique window into the complex interplay between the specialized signaling center AER and other limb development–related cell populations. Our recombinant budoid setup allows precise manipulation of cell numbers and compositions, enabling systematic assessment of how these variables influence 3D morphogenesis. This controlled, cell sorting–based approach supports reproducible spatial patterns and quantitative analyses that are not easily achievable in vivo. Taking advantage of this platform, we have uncovered that mesodermal cells have the capacity to self-organize and break symmetry upon brief FGF8b treatment, independently of any 3D embedding (e.g., Matrigel), and that even a transient and small number of AER-like cells can exert morphological, spatial, and molecular changes on mesodermal cell fates. Critically, complementing an earlier tissue- and gene-based study (*2*), our findings quantitatively show that the impact of specialized signaling center cells is not only limited to nearby cells but can extend to spatially distant populations, consequently shaping tissue organization.

Although our initial objective was to generate only signaling center AER cells, our protocol unexpectedly produced self-organizing heterogeneous cultures with limb development properties. While the exact mechanisms underlying the simultaneous formation of limb development–related populations remain unclear, including whether some cells transiently form an intermediary coelomic epithelium, our protocol enabled us to systematically study individual limb populations beyond signaling center AER cells and to develop a simplified mesodermal organoid model capturing aspects of limb morphogenesis. In contrast to earlier limb models (including a limb bud organoid–like model) (*9*–*13*), our protocol eliminates the need for manual manipulation of organoids and achieves 3D morphogenesis without embedding (e.g., Matrigel), which can be both costly and inconsistent. Using a simple 12-well plate setup, we can efficiently generate thousands of budoids in a single experiment—a scale unprecedented in the study of limb development within a 3D framework. This scalability supports diverse experimental approaches for exploring limb developmental processes. In addition, our comprehensive single-cell analyses, in vivo comparisons, and quantitative spatial gene expression profiling allowed us to characterize the molecular and cellular properties of each population, demonstrating that our organoids reliably elicit 3D morphogenesis with high reproducibility and certain limb developmental features. Compared to previously used micromass cultures (*34*) and protocols to generate articular chondrocytes (*35*), budoids express similar lineage markers for chondrocytes, tenocytes, and related mesodermal derivatives, yet do so in a multilineage context with ectoderm-mesoderm interactions in 3D and emergent spatial organization. These advances, along with the feasibility, efficiency (~80% of aggregates exhibiting chondrogenesis-based symmetry breaking), and affordability of our method, position budoids as a multipurpose and easily adoptable method for a wide range of studies, accessible to many laboratories.

Pseudostaging comparisons with in vivo limb datasets suggest that some budoid cell types resemble early limb stages (e.g., Mes_Hoxb day 9 samples for E9.5), while others are more similar to later stages (e.g., Mes_Sox9 day 9 for E12.5) (fig. S8B), resulting in an apparent mixed stage profile. We note that such patterns may, in part, reflect differences in cell type annotations across datasets (specifically for mesodermal populations) and technical biases in single-cell capture, particularly the underrepresentation of ectodermal cells, rather than true biological heterogeneity. Moreover, there is currently no consensus on mesodermal cell types in the developing limb, and many scRNA-seq datasets annotate clusters arbitrarily without demonstrating their functional roles [e.g., (*22*, *26*, *36*–*39*)]. This ambiguity complicates the interpretation of predicted cellular interactions in a biologically meaningful way. Resolving the developmental staging and precise cellular identities of budoids with greater precision will require not only improved reference datasets but also advances in single-cell methodologies that minimize the distortion of tissue cellular composition.

Budoids reproducibly form two polarized domains, with cartilage consistently forming at one end and the opposite domain expressing genes associated with limb bud mesoderm and soft connective tissue lineages. We refer to the former as the “proximal side” in alignment with the distal-to-proximal pattern of cartilage differentiation observed in vivo. Whether budoids display further axial patterning beyond this polarized differentiation and spatially restricted domains that mirror in vivo organization remains to be determined. Notably, Hox gene expression was low in 2D cultures (fig. S3D), and its activation became more apparent only under 3D conditions. The reasons for this remain unclear and may reflect effects of prolonged culture, exposure to specific morphogens (e.g., FGF8), or the 3D architecture itself.

It is important to note that we do not propose budoids to be equivalent to limb buds but rather simplified 3D organoid models that can be used to examine epithelial signaling center-mesoderm interactions and that recapitulate specific aspects of limb morphogenesis (e.g., mesoderm-to-cartilage differentiation and AER-mediated signaling). As with other organoid systems, budoids are not intended to fully replicate the organ or structure itself but instead serve as experimentally tractable models to study certain developmental processes (as indicated above). While in vivo studies remain the gold standard for understanding limb morphogenesis, they are limited in scale, throughput, and the ability to apply repeated or combinatorial perturbations to signaling centers. By contrast, our budoid system enables parallel generation of hundreds of structures, supporting large-scale, quantitative, and reproducible analyses. This scale allows statistical robustness, integration of multiplexed spatial and transcriptomic data, and systematic perturbations of morphogen gradients and cell composition—approaches impractical in embryos. Although in vitro models may lack complete organ specificity, preserving key cellular players, signaling interactions, and morphogenetic processes makes them highly informative for probing mechanisms otherwise inaccessible in vivo.

A key distinction of budoids (whether derived from stem cells or in vivo E12.5 limbs) compared to native limb buds is the internalization of ectodermal cells, which would normally envelop the mesoderm. This behavior is partly consistent with Steinberg’s differential adhesion hypothesis (*40*), where cells sort on the basis of differences in adhesion. However, the mechanisms underlying this internalization in budoids remain unclear, including whether they parallel findings in other systems [e.g., *Xenopus* (*41*, *42*), zebrafish (*29*), and *Hydra* (*43*)]. Moreover, both our 2D domes and budoid systems may capture more general features of ectoderm-mesoderm interaction–based bud formation seen across organ systems.

The consistency of budoids across experiments provides a robust foundation for systematic improvements and functional testing. Analogous progress in other organoid systems—such as gastruloids—further highlights the potential for refinement through iterative optimization. Gastruloids, which model early embryonic development (*44*), have provided valuable insights and incorporated additional features like somite formation through subsequent modifications (*45*). Similarly, potential improvements to budoids could focus on characterizing long-term budoids and achieving the ectodermal coverage of the mesoderm, enabling a more accurate representation of limb morphogenesis and enhancing its utility as a model system. Improvements to the budoid system could make it more comparable to explants, representing an ideal goal in terms of reproducibility and extended culture duration.

Our study highlights the importance of highly controlled and scalable cellular assays, integrating cell sorting, recombinant budoids, morphological analyses, and sequencing- and hybridization-based assessments to investigate morphological features, gene expression changes, and cell type formation. These findings align with the established role of AER-like cells in guiding mesodermal cells toward in vivo–like lineages. While in utero grafting of these cells onto mouse embryonic limbs and evaluating their contribution to development offer a technically challenging yet valuable method for further confirmation of in vivo–like properties, additional refinements are necessary. Specifically, our generated AER-like cells face challenges in aggregating, and limb bud mesodermal cells require exogenous inputs for successful aggregation. Future advancements, such as coaxing cells with extracellular matrix components and characterizations, will be of value.

Our stem cell–based protocols can lead to the mechanistic and quantitative understanding of specialized signaling centers and their interactions with mesodermal cells, which can also guide regenerative therapies. Bioengineering strategies have been used to emulate single or multiple morphogen gradients (*46*, *47*), and our generated in vivo–like specialized signaling center cells could enable the dissection of the matrix of signaling gradients and cell-cell interactions, potentially also improving endeavors of computational limb modeling (*48*). Furthermore, our protocols can be adapted to stem cells from other species, including human, for comparative analyses (*49*). Last, the scalability of budoids paves the way for high-throughput genetic and chemical screens to identify factors that promote cartilage formation for therapeutic purposes.

Efforts to regenerate mammalian limbs or digits have focused on transplanting the limb bud mesoderm with limited success (*9*, *50*). Meanwhile, the effectiveness of specialized signaling centers (*51*), and particularly AER cells (*52*, *53*), has been emphasized in amphibian regeneration. Therefore, our methods robustly forming specialized signaling center AER-like cells and other limb cell types could be foundational in fundamental regeneration research, as well as transplantation studies aimed at inducing blastema for mammalian regeneration. On this basis, several important areas for improvement remain. First, our protocol produces newly forming AER-like cells, awaiting identification of factors to maturate them. This can also inform how AER is regulated in vivo, as well as examine their possible formation following limb amputations. Second, our functional assays demonstrate that AER-like cells are hard to maintain and show considerable aggregation problems, which effectively limits transplantation studies. Last, although we were able to FACS-enrich specific populations, refinements to our protocol will increase their purity. Because of the highly efficient nature of our method, we anticipate that future studies can systematically address these challenges.

In summary, we provide a new methodology for studying specialized signaling centers and their interactions with the mesoderm in a simplified setting. Our methodology could lead to an in-depth and quantitative understanding of development, congenital disorders, cross-species features, and new directions for regenerative medicine.

## MATERIALS AND METHODS

### mESC culture

129/SvEv mESCs (a gift of D. Duboule) were maintained in six-well plates coated with 0.1% gelatin (Merck, ES-006-B, and Stem Cell Technologies, 07903, used interchangeably) in serum + 2i/Lif [Dulbecco’s modified Eagle’s medium (DMEM), high glucose, GlutaMAX supplement (Gibco, 61965-026)] supplemented with 10% embryonic stem cell fetal bovine serum (FBS; Gibco, 16141-079), 1× nonessential amino acids (Gibco, 11140035), 1 mM sodium pyruvate (Gibco, 11360-039 or 11360070), 0.1 mM 2-mercaptoethanol (Gibco, 31350-010), penicillin/streptomycin (100 U/ml; Gibco, 15140122), LiF (100 ng/ml; produced by the Protein Production and Structure Core Facility at EPFL), 1 μM PD0325901(Sellechckem, S1036), and 3 μM CHIR99021 (ApexBio (A3011) or Calbiochem (361559) used interchangeably. Media were changed every 2 days, or cells were passaged every 2 to 4 days. Cells were grown in an incubator at 37°C and 5% CO_2_. Routine mycoplasma tests were performed.

### Plasmid construction and cell line establishment

A CRISPR-Cas9 approach was used to generate the *Fgf8* reporter line. Annealed oligos encoding guide RNAs targeting near the *Fgf8* stop codon (the forward oligo 5′-caccGAGCGCCTATCGGGGCTCCG-3′ and the reverse oligo 5′-aaacCGGAGCCCCGATAGGCGCTC-3′) were ordered from IDT and ligated into a pX330 vector digested with Bbs I, as described previously (*54*). The 5′ homology arm [800 base pairs (bp)] and 3′ homology arm (800 bp) flanking from the *Fgf8* stop codon were amplified by polymerase chain reaction (PCR) from mouse genomic DNA and cloned into the homology-directed repair donor vector containing tdTomato and puromycin resistance gene (a gift of N. Irie) (*55*) using in-fusion cloning (Clontech) according to the manufacturer’s recommendations. The cloned plasmid was verified by Sanger sequencing. For knockin, 20,000 mESCs were seeded in 24-well plates coated with 0.1% gelatin. One day after seeding, mESCs were transfected with a donor vector and guide RNA–containing pX330 vector using 2.4 μl of Lipofectamine 2000 (Invitrogen ref. 11668-019) per 800 ng of total plasmids according to the manufacturer’s recommendations. Puromycin selection was applied. The remaining cells were passaged, and 1000 cells were seeded on 60 mm–by–15 mm plates coated with 0.1% gelatin for colony picking. Grown single colonies were picked manually, expanded, and genotyped by PCR. Genomic DNA was first extracted from cell pellets in lysis buffer [10 mM tris, pH 8.0, 100 mM NaCl, 10 mM EDTA, and 0.5% SDS; proteinase K (PK) was added immediately before lysis at a final concentration of 0.2 mg/ml] at 56°C for 4 hours to overnight, followed by PK inactivation at 98°C for 10 min (*55*). The supernatant was used directly in genotyping PCR. PCR-verified clones were expanded, and genomic DNA from the expanded line was used to confirm integration by Sanger sequencing. Primers used for genotyping are shown in data S4. No leaky expression of *TdTomato* was observed in mESCs.

PiggyBac (PB) transposase–based integration was used to generate *CAG:mVenus* and *Prrx1^Enh^:mVenus* mESC lines. The PB vector containing *CAG:mVenus* was provided by N. Irie. *Prrx1^enh^* (*24*) is cloned from mouse genomic DNA into the PB vector using In-Fusion (Takara) according to the manufacturer’s recommendations. The cloned plasmid was verified by sequencing. For integration, 20,000 mESCs were seeded in gelatinized 24-well plates. One day later, cells were cotransfected with one of the PB vectors, puromycin resistance plasmid (a gift of D. Suter), and PB transposase plasmids using 2.4 μl of Lipofectamine 2000 (Invitrogen ref. 11668-019) per 800 ng of total plasmids according to the manufacturer’s recommendations. Puromycin selection was performed. No leaky expression of *Prrx1^Enh^:mVenus* was observed in mESCs.

### Heterogeneous culture induction

The mESCs were washed with 1× phosphate-buffered saline (PBS; Gibco, 10010015), dissociated with 1× 0.25% trypsin-EDTA (Gibco, 25200072) for 2 min at 37°C, and deactivated in mESC media without inhibitors (2i/Lif). Cells were collected in 15-ml Falcon tubes containing 8 ml of 1× PBS (to dilute the remaining factors further) and centrifuged at 137 relative centrifugal force (rcf) for 5 min at room temperature (RT). The media were aspirated, and cells were resuspended to single cells in NNE media [Glasgow’s minimum essential medium (Gibco, 11710-035) supplemented with 1.5% knockout serum replacement (Gibco, 10828010), 1× nonessential amino acids (Gibco, 11140050), 1 mM sodium pyruvate (Gibco, 11360-039 or 11360070), 0.1 mM 2-mercaptoethanol (Gibco, 31350-010), and normocin (0.1 mg/ml; InvivoGen, ant-nr-1) final concentrations] and counted. A total of 40,000 cells were seeded in 12-well plates coated with 0.1% gelatin and incubated at 37°C in 5% CO_2_. On day 1 after seeding, the cells were washed with 1× PBS, and the medium was changed to fresh NNE medium (1 ml per well). On day 3, high cell death was observed. The medium was aspirated, and the cells were washed twice with 1× PBS and changed to 1 ml of fresh NNE containing SB431542 (Apexbio, A8249) at a 1 μM concentration and BMP4 (100 or 200 ng/ml; PeproTech, 120-05ET) final concentrations. On day 5, the cells were washed twice with 1× PBS, and the medium was changed to 1 ml of fresh NNE containing FGF10 (100 ng/ml; PeproTech, 100-26), BMP4 (100 or 200 ng/ml; PeproTech, 120-05ET), and 4.5 μM CHIR99021 (Apex Bio) or 3 μM CHIR99021 (Calbiochem) final concentrations. Medium changes were performed at 48-hour intervals (±2 hours). All recombinant proteins and chemicals used in cell culture were reconstituted in the buffers as recommended by the supplier. During this study, we detected batch variation for CHIR99021 and recombinant BMP4. Most experiments in this study were performed with 4.5 μM CHIR99021 (Apex Bio), and all experiments (except 2D culture scRNA-seq experiments) were replicated with 4.5 μM CHIR99021 (Apex Bio). Similarly, most experiments in this study were performed with BMP4 (100 ng/ml; PeproTech, 120-05ET), and all experiments were replicated with BMP4 (100 ng/ml; PeproTech, 120-05ET).

### Quantitative RT-PCR

For quantitative reverse transcription PCR (qRT-PCR) analysis, total RNA was purified using the RNeasy Mini Kit (Qiagen, 74106) for day 7 samples and the PicoPure RNA isolation kit (Thermo Fisher Scientific, KIT0204) for sorted samples according to the manufacturer’s protocol for both kits. One microgram of RNA was used for cDNA synthesis using SuperScript II (Invitrogen, 18064-014). qRT-PCR was performed with Power SYBR Green PCR Master Mix (Applied Biosystems, 4367659) using the QuantStudio 6 or 7 Flex Real-Time PCR Systems (Applied Biosystems), and data were calculated using the delta-delta *C*_t_ method. Rpl27 was used as a housekeeping gene in all experiments. In fig. S1 (E and L), the relative expression of each gene at day 7 was calculated relative to the mESCs (day 0) used in the same experiment. In fig. S9 (D and E), the relative expression of sorted populations was calculated relative to the mRNA extracted from one of the mESC (day 0) data. Hamilton Microlab Star (Hamilton) was used to assemble the plates. Only two technical replicates were used for each sample, as the Hamilton Microlab allows precise pipetting. The primers used in qRT-PCR are shown in data S4.

### Generation of budoids in 3D cultures from mESCs

To generate budoids, the first 7 days followed the induction protocol as described above. On day 7, cells were dissociated with 0.25% trypsin-EDTA (Gibco, 25200072) for 4 to 5 min at 37°C and deactivated with mESC media without inhibitors (2i/Lif). Cells were collected in a 15-ml Falcon tube containing 8 ml of 1× PBS, centrifuged at 400 rcf for 5 min, resuspended in NNE medium, and counted. A suspension (90,000 cells/ml) was prepared with NNE medium containing WNT3a (300 ng/ml; PeproTech, 315-20) and FGF8b (450 ng/ml; PeproTech, 100-26), and 100 μl of cell suspension per well was seeded into 96-well low-attachment plates (Thermo Fisher Scientific, 174929). Please note that in the experiments for fig. S5, different concentrations of these recombinant proteins were tested. For the BMP pathway perturbation experiments described in Fig. 3 (I and J), LDN193189 (MedchemExpress, HY-12071A-10MM/1ML), recombinant NOGGIN (PeproTech, 120-10C), or recombinant BMP4 (PeproTech, 120-05ET) was added at specified concentrations during budoid generation. In these experiments, the final concentration of LDN193189, NOGGIN, or BMP4 was maintained throughout the experiments. DMSO (dimethyl sulfoxide; 0.4%) was used as a vehicle control. No reservoirs were used to seed the cells to eliminate the possibility of WNT3a adhering to the reservoir surface. Fifty microliters of the media was changed once every 48 hours (±2 hours), and samples were collected at the reported time points. Cells were grown in an incubator at 37°C and 5% CO_2_. The outer layer of the 96-well plates was not used during budoid generation and was filled with 100 μl of 1× PBS to avoid side effects as a result of evaporation.

### Generation of budoids from in vivo E12.5 mouse limbs

Mouse embryo samples were collected in accordance with the Swiss Federal Veterinary Office guidelines and as authorized by the Cantonal Veterinary Office (cantonal animal license no: VD3652c; national animal license no: 33237). Pregnant female CD1 mice were purchased from Charles Rivers Laboratories, and E12.5 embryos were collected by dissecting the uterine horn. The hindlimbs and forelimbs of embryos were collected and dissected for proximal and distal regions, as shown in fig. S6A. No distinction was made between left or right hindlimbs or forelimbs, and no sex determination was done. Samples were collected in tubes containing 1× PBS, each containing 10 to 15 samples. Samples were washed once with 1× PBS, dissociated with 0.25% trypsin-EDTA (Gibco, 25200072) for 5 min at 37°C, and deactivated with DMEM (with 10% FBS). Cells were collected and centrifuged at 400 rcf for 5 min and resuspended in NNE medium containing no additives, FGF8b (450 ng/ml; PeproTech, 100-26), or WNT3a (300 ng/ml; PeproTech, 315-20) and FGF8b (450 ng/ml; PeproTech, 100-26). One hundred microliters of cell suspension per well was seeded into 96-well low-attachment plates (Thermo Fisher Scientific, 174929). Fifty microliters of the media was changed once every 48 hours (±2 hours), and samples were collected at the reported time points. Cells were grown in an incubator at 37°C and 5% CO_2_. All recombinant proteins and chemicals used in the cell culture were reconstituted in the buffer as the supplier recommended.

### MOrgAna (machine-learning organoid analysis)–based area and elongation analysis and mVENUS-based polarization quantification

MOrgAna (machine-learning organoid analysis) (*56*) was used to automate the area and elongation phenotypes of the generated 3D structures. A total of 5% of the generated data were used to train MOrgAna by manual masking for each different experimental design. MOrgAna outputs Excel files with the area and major and minor axis lengths. For elongation, a set of structures was manually examined and classified into three categories: not elongated, elongated (short), and elongated (long) by two independent researchers. These classifications were compared to the major and minor axis measurements obtained from MOrgAna. The ratio of minor axis length to major axis length was taken, and thresholds were determined that resulted in minimal classification errors for each category. Values ≥0.85 correspond to no elongation, 0.75 to 0.85 to (short) elongation, ≤0.75 to (long) elongation. To quantify polarized mVenus+ cells in Fig 4G, a bright-field image overlaid with fluorescence images of recombinant budoids having a clearmVenus signal was used, and the number of samples showing polarization was manually counted.

Enriched cells were counted and processed as described in the regular budoid generation protocol (with 90,000 cells/ml), with or without FGF8b (450 ng/ml) treatment, as indicated in the relevant figures. For recombinant budoid experiments, individual enriched populations were mixed in separate tubes at a 1:1 ratio, totaling 90,000 cells/ml with or without the addition of FGF8b (450 ng/ml), as indicated in the relevant figures. One hundred microliters of the solution was seeded into 96-well low-attachment plates (Thermo Fisher Scientific, 174929). Images were taken 1 (day 8 of the full protocol) and 7 (day 14 of the full protocol) days after seeding via Nikon Eclipse Ti2.

### FACS and flow cytometry–based quantification

For antibody labeling–based ectodermal cell sorting experiments, the CAG:mVENUS mESCs line was used to sort surface ectoderm–like (CD9^High^/EPCAM^High^) or AER-like (CD9^Low^/EPCAM^High^) cells. Day 7 induction cells were washed once with 1× PBS, dissociated with 0.25% trypsin-EDTA (Gibco, 25200072) for 5 min at 37°C, and deactivated with DMEM (with 10% FBS). Cells were collected, centrifuged at 400 rcf for 5 min, and resuspended in cold FACS buffer [1× PBS supplemented with 1% bovine serum albumin (BSA; Gibco, 15260037)]. Cells were then stained for the surface markers EpCAM (Invitrogen, 25-5791-80) and CD9 (Invitrogen, 12-0091-81) in FACS buffer for 45 min on ice in the dark. The cells were then washed with 1 ml of ice-cold FACS buffer and centrifuged at 600 rcf for 5 min, with the washes repeated a total of two times. Cells were then resuspended to a single-cell suspension in a cold FACS buffer in a round-bottom polystyrene tube, with a cell strainer (Corning, 352235), and stored in the dark until analysis/cell sorting. For EpCAM staining–based mesoderm enrichment (EpCAM^Neg^), wild-type mESCs were used with the above protocol, except that CD9 staining was not performed during labeling. For *Fgf8:tdTomato*-based cell sorting, the same induction and dissociation protocols were repeated with the *Fgf8:tdTomato* mESC line. Dissociated cells were resuspended to a single-cell suspension in cold FACS buffer in a round-bottom polystyrene tube, with a cell strainer, and stored in the dark until analysis/cell sorting. FACSAria Fusion and Sony SH800 were used for cell sorting. Fig. S9 (B, C, and F) presents example gating strategies for cell sorting. FACS experiments were also used to quantify EpCAM+ and *Fgf8:tdTomato*+ cell numbers, as reported in Fig. 1 (E and F). Fig. S1 (J and K) presents examples of flow cytometry–based quantifications and gating. In all experiments, an unstained wild-type control was used to label negative cells, and single staining was performed for compensation when necessary. FlowJo was used for further analysis.

### Individual lineage and recombinant budoid experiments

The above-described cell sorting experiments were performed to enrich individual lineages. Enriched cells were counted and processed as described in the regular budoid generation protocol (with 90,000 cells/ml), with or without FGF8b (450 ng/ml) treatment, as indicated in the relevant figures. For recombinant budoid experiments, individual enriched populations were mixed in separate tubes at a 1:1 ratio, totaling 90,000 cells/ml with or without the addition of FGF8b (450 ng/ml), as indicated in the relevant figures. One hundred microliters of the solution was seeded into 96-well low-attachment plates (Thermo Fisher Scientific, 174929). Images were taken 1 (day 8 of the full protocol) and 7 (day 14 of the full protocol) days after seeding via Nikon Eclipse Ti2.

### Immunostaining and hybridization chain reaction (HCR)

For immunostaining in 2D cultures, the induction protocol was performed in 12-well glass-like polymer coverslip-bottom plates (Cellvis P12-1.5P). On day 7, samples were washed with 1× PBS and fixed with 4% formaldehyde (FA; Thermo Fisher Scientific,119690010) for 10 to 12 min at RT. This was followed by washing samples with 1× PBS for 2 × 5 min. To permeabilize cells, samples were incubated with 0.1% Triton X-100 (Sigma-Aldrich) in 1× PBS (hereafter, PBS-T) for 3 × 10 min. The samples were then incubated with a blocking solution [50% Cas-Block (Thermo Fisher Scientific, 008120) and 50% PBS-T] for 30 min. The samples were then incubated with primary antibodies [TP63 (Abcam, ab735), TFAP2C (Cell Signaling, 2320S), LEF1 (Abcam, ab137872)], diluted in blocking buffer overnight at 4°C. To enhance the reporter signal, suitable samples were stained with anti–GFP (green fluorescent protein) (Abcam, ab13970) (for Prrx1 enhancer reporter experiments) or anti–RFP (red fluorescent protein) [a gift from S. Pons (*57*)] (for Fgf8 reporter experiments). The next day, samples were incubated with 1× PBS-T for 3 × 10 min. Samples were then blocked by a blocking solution for 30 min, followed by incubation with a blocking solution containing Alexa Fluor 488 (AF-488; Thermo Fisher Scientific, A11039 and A21202)–, AF-594 (Thermo Fisher Scientific, A11012)–, and/or AF-647 (Thermo Fisher Scientific, A32728)–conjugated secondary antibodies specific for the host species of the primary antibodies for 1 hour at RT in the dark. During secondary antibody incubations, phalloidin staining was done by adding the relevant material (Abcam, ab176757 and ab176753) for relevant experiments. After secondary antibody incubations, samples were washed with PBS-T for 3 × 10 min and then with 1× PBS for 3 × 10 min. Samples were incubated in 1× PBS with Hoechst 33342 (Sigma-Aldrich, 2261) for 15 min to stain for nuclei and then were washed with 1× PBS. The protocol was performed at RT, unless otherwise stated. The samples were stored at 4°C in the dark (up to 2 weeks) before imaging on a Leica SP8 inverted confocal microscope. All incubations were performed on a gentle rotation system.

For whole-mount budoid immunostaining, budoids were collected from 96-well plates using low binding tips (Sigma-Aldrich, Z719668), with a cut tip, and handled with low binding tips throughout the protocol. Collected budoids were washed with 1× PBS and fixed with 4% FA (Thermo Fisher Scientific,119690010) in 1× PBS at RT. This was followed by incubating budoids with 1× PBS for 10 min. To permeabilize samples, budoids were incubated with PBS-T for 3 × 10 min. Budoids were then incubated in a blocking solution [50% Cas-Block (Thermo Fisher Scientific, 008120) and 50% PBS-T] for 30 min. Budoids were then incubated with primary antibodies [TP63 (Abcam, ab735), SOX9 (Merck, AB5535), and E-CADHERIN (BD, 610405)] diluted in a blocking buffer overnight at 4°C. The next day, budoids were incubated in PBS-T for 3 × 10 min, reblocked with a blocking solution for 30 min, and incubated with a blocking solution containing AF-488 (Thermo Fisher Scientific, A32731)– and/or AF-647 (Thermo Fisher Scientific, A32728)–conjugated secondary antibodies specific for the host species of the primary antibodies for 1 hour at RT in the dark. After secondary antibody incubations, budoids were washed with PBS-T for 3 × 10 min and then with 1× PBS for 3 × 10 min. Budoids were incubated in 1× PBS with Hoechst 33342 (Sigma-Aldrich, 2261) for 15 min at RT to stain for nuclei and then were washed with 1× PBS. The samples were stored at 4°C in the dark (up to 2 weeks) before imaging on a Leica SP8 inverted confocal microscope. Please note that for E-cadherin or TP63 staining, samples were fixed with 4% FA for 20 min and overnight, respectively, and the same above protocol was applied. All incubations were performed on a gentle rotation system to prevent potential buckling of budoids. The protocol was performed at RT, unless otherwise stated.

Hybridization chain reaction (HCR) (*58*) was performed as described previously for 2D cultures and with modifications for budoids, and materials for HCR, including probes, were purchased from Molecular Instruments. Budoids were collected from 96-well plates using low binding tips (Sigma-Aldrich, Z719668) with a cut tip and handled with low binding tips throughout the protocol. Budoids were washed with 1× PBS and then fixed with 4% FA (Thermo Fisher Scientific, 119690010) in 1× PBS for 30 to 60 min. Samples were permeabilized in 70% ethanol for 1 hour and collected in Eppendorf tubes. The supernatant was removed, washing solution was added, and samples were rotated for 10 min. The supernatant was removed and replaced with hybridization buffer for 30 min of incubation at 37°C. In parallel, a probe solution was prepared by diluting mRNA targeting probes to 30 to 40 nM in 200 μl of hybridization buffer and incubated at 37°C for 30 min. The hybridization buffer was removed from the samples, and the probe solution was applied to the samples for 12 to 16 hours of incubation at 37°C. The samples were then washed twice for 10 min with wash buffer and twice for 20 min with 5× SSC with 0.1% Tween 20 (SSC-T) at RT. To visualize the probes, an amplification solution was prepared by first heating the pairs of fluorophore-tagged hairpins (h1 and h2 hairpins) corresponding to the probes to 95°C for 90 s. The hairpins were then left in the dark at RT for 30 min. The final amplification solution was prepared at 40 to 60 nM h1 and h2 in 200 μl of amplification buffer. Samples were first incubated in an amplification buffer without hairpins for 10 min and then placed in the final amplification solution at RT, protected from light, for 12 to 16 h. Samples were washed for 2 × 20 min with 5× SSC-T. The samples were stained with 1× PBS with Hoechst 33342 (Sigma-Aldrich, 2261) and washed in 1× PBS for 10 min. Stained budoids were transferred to imaging plates and stored at 4°C in the dark (up to 2 weeks) before imaging on a Leica SP8 inverted confocal microscope. These procedures were performed at RT, unless otherwise stated.

In fig. S1F, 2D cultures were first processed with HCR and then with immunostaining as described above with minor modifications. Briefly, samples were processed with HCR [as reported before (*58*)]. Then, the samples were incubated with a blocking solution [50% Cas-Block (Thermo Fisher Scientific, 008120) and 50% PBS-T] for 30 min. The samples were then incubated with a TP63 primary antibody (Abcam, ab735) diluted in blocking buffer overnight at 4°C. The next day, samples were incubated with 1× PBS-T for 3 × 10 min. Samples were then blocked by a blocking solution for 30 min, followed by incubation with a blocking solution containing AF-680 (Thermo Fisher Scientific, A32729)–conjugated secondary antibodies for 1 hour at RT in the dark. After secondary antibody incubations, samples were washed with PBS-T for 3 × 10 min and then with 1× PBS for 3 × 10 min. Samples were incubated in 1× PBS with Hoechst 33342 (Sigma-Aldrich, 2261) for 15 min to stain for nuclei and then were washed with 1× PBS. The protocol was performed at RT, unless otherwise stated. The samples were stored at 4°C in the dark (up to 2 weeks) before imaging on a Leica SP8 inverted confocal microscope.

For immunostaining recombinant budoids in Fig. 4 and fig. S11, flash-frozen optimal cutting temperature–embedded samples were used. Please note that the subsequent sections of HybISS samples were used in these experiments. The sections were allowed to acclimate to RT and fixed with 4% paraformaldehyde for 15 min (Electron Microscopy Sciences, 15710-S). This was followed by one wash in 1× PBS, and the samples were incubated in PBS-T for 3 × 10 min. The samples were then incubated with a blocking solution for 1 hour at RT, and they were then incubated with primary antibodies [SOX9 (Merck, AB5535) and TWIST1 (Abcam, ab50887)] diluted in a blocking buffer overnight at 4°C. To enhance the GFP signal, suitable samples were stained with anti-GFP (Abcam, ab13970). The next day, they were washed three times with the wash buffer (0.1% Triton X-100 in PBS) and incubated with a blocking solution [50% Cas-Block (Thermo Fisher Scientific, 008120) and 50% PBS-T] for 15 min. Then, the sections were incubated with AF-488 (Thermo Fisher Scientific, A11039)–, AF-594 (Thermo Fisher Scientific, A11012)–, and/or AF-647 (Thermo Fisher Scientific, A32728)–conjugated secondary antibodies specific for the host species of the primary antibodies for 1 hour at RT in the dark. After three washes in wash buffer and then in 1× PBS, sections were mounted with either SlowFade Gold Antifade Mountant with 4′,6-diamidino-2-phenylindole (DAPI; Invitrogen S36939) or VECTASHIELD antifade mounting medium with DAPI (VectorLabs, H-1200-10). The samples were stored at 4°C in the dark (up to 2 weeks) before imaging on a Leica SP8 upright confocal microscope. Images were analyzed using Fiji software.

### Confocal and bright-field imaging

Bright-field images in Fig. 2D and figs. S1 (A to D), S5 (B and E), and S6E were taken with Olympus CKX53SF. Bright-field images in fig. S6A were taken with Nikon Stereo Microscope SMZ745T KL 300 LED (light-emitting diode) light source. Bright-field and fluorescence images in Figs. 2G and 4 and fig. S10 were taken with Nikon Eclipse Ti2. Confocal images in Figs. 1 and 3 and figs. S4 and S8 were taken with a Leica SP8 inverted confocal microscope with 10×/0.30 HC PL Fluotar or 20×/0.75 HC PL APO objectives. Confocal images in Fig. 4F and fig. S11 were taken with a Leica SP8 upright confocal microscope with 20×/0.75 HC PL APO or 40×/1.25 HC PL APO objectives. For the confocal images, LAS X was used to set up tiled images, and a 10 to 20% overlap between tiles was used. All images were analyzed using Fiji software. Fiji was used to maximize the projection of the *z*-stacks and to adjust the contrast to emphasize biological relevance. When necessary, images were cropped, flipped, and/or rotated to highlight biological relevance.

### SOX9 distribution quantification in budoids

Maximum projections for *z*-stack images of organoids were manually masked in Fiji, and fluorescence intensity was calculated across the organoid major axis using the built-in intensity plot profile tool. The gray values and length of each organoid were normalized to values between 0 and 1. Averaged intensity profiles were generated by bucketing using 200 bins for the organoid length at increments of 0.005.

### Sample preparation for scRNA-seq

The 2D induction and budoid generation were performed as described above, and samples were collected at designated time points. In the first replicate of 2D cultures, samples were collected during days 0, 3, 5, and 7 of the protocol, dissociated with 0.25% trypsin-EDTA (Gibco, 25200056) for 5 min at 37°C, and deactivated with mESC media without inhibitors (2i/Lif). Cells were collected, dissociated and centrifuged at 400 rcf for 5 min, resuspended in 1× PBS supplemented with 1% BSA (Sigma-Aldrich, A9418-10G), and filtered through a strainer. Samples were multiplexed using CellPlex, according to the manufacturer’s recommendations, and scRNA-seq libraries were generated using 10× Genomics (v3 chemistry) and sequenced in pools of two samples per lane on an Illumina HiSeq 4000, with the parameters 28 bp (read 1), 8 bp (i7 index), and 91 bp (read 2), as per standard 10× Genomics recommendations. In the second replicate of 2D cultures, only day 5 and 7 samples were collected and processed in the same way and sequenced on NovaSeq 6000 with parameters: 28 bp -read 1; 8 bp - i7 index; and 91 bp - read 2. For budoids sequencing experiments, the first experiment contained 60 pooled D12 elongated budoids, and processed with CellPlex as described above. The second experiment contained 60 pooled D9 budoids and 60 elongated D12 budoids. Briefly, individual budoids were collected from 96-well plates with a low attachment tip with a cut tip, and washed with 1X PBS, dissociated with 0.25% trypsin-EDTA (Gibco, 25200072) for 10 minutes at 37°C with shaking at 300 rpm, deactivated with mESCs media without inhibitors (2i/Lif). Cells were collected, centrifuged at 600 rcf for 5 minutes at 4°C, resuspended in 1X PBS supplemented with 0.04% BSA (Sigma, A9418-10G), filtered and counted. Then, scRNA-seq libraries were generated and sequenced. These samples were processed and sequenced on NovaSeq 6000 with parameters 28 bp (read 1), 10 bp (i7 index), and 90 bp (read 2). Both replicates of 2D cultures and the first replicate of budoids received 3 μM CHIR99021 (Calbiochem) on day 5 of the induction, and the second replicate of budoids received 4.5 μM CHIR99021 (ApexBio) on day 5 of the induction; the results showing similarity across replicates are shown in fig. S7C.

### Analysis of scRNA-seq

Each experiment was processed separately with CellRanger (version 7.1.0) to obtain the expression matrix. Cells were filtered with dataset-specific thresholds on the basis of mitochondrial percentage and the number of unique molecular identifiers and genes (data S1). The expression matrix was normalized. Technical confounding factors, including cell cycle effect and sequencing depth, were regressed out with the ScaleData function in Seurat (version 4.3.1) (*59*).

Seurat reciprocal PCA–based integration was used to perform the coclustering of cells from different experiments. In the integration of the 2D cells (days 0, 3, 5, and 7) from experiments 1 and 2 (Fig. 1G and fig. S2), genes were ranked by the number of datasets they are deemed variable (*SelectIntegrationFeatures*), and the top 3000 genes were used to integrate all the datasets (*FindIntegrationAnchors* and *IntegrateData*). The first 30 principal components (PCs) were chosen to build a K nearest neighbors (KNN) graph and clustering. Cell type annotation was based on the expression of literature-supported cell type markers. Uniform manifold approximation and projection (UMAP) was performed for visualization. The default setting was used unless noted. Similarly, cells from days 9 and 12 from budoid sequencing experiments 1, 2, and 3 were integrated (Fig. 3B and fig. S7). Data were normalized using SCTransform, where the cell cycle effect and sequencing depth were regressed out. The final UMAP was generated using the first 40 PCs.

### Transcriptome-wide comparison of cluster similarity between in vivo and in vitro cells

ClusterFoldSimilarity (*60*) was used to calculate the cluster similarity between in vivo tissue and our in vitro system (figs. S3B and S8A). ClusterFoldSimilarity scores cluster similarity by comparing the fold-change patterns in clusters that share a common set of genes. The top 3000 highly variable genes identified by *Seurat::FindVariableFeatures* were used to calculate the similarity. Limb bud mesoderm subtypes were annotated on the basis of a published comprehensive atlas (*22*)

### Projection of in vitro scRNA-seq datasets onto the published in vivo reference

The reference dataset with annotation was downloaded from https://limb-dev.cellgeni.sanger.ac.uk/ (*22*). Major ectodermal and mesodermal cell types were kept, including *AER-Basal*, *Adh + Fibro*, *Basal*, *DermFibro*, *DistalMes*, *Dpt + Fibro*, *EarlyDistalMes*, *EarlyProxMes*, *Hoxc5 + DermFibroProg*, *HyperChon*, *InterMusFibro*, *InterZone*, *Meox2 + Mes*, *MesCond*, *Mfap5 + Fibro*, *OsteoB*, *PrehyperChon*, *ProlifChon*, *ProxMes*, *Rdh10 + DistalMes*, *RestingChon*, *Teno*, and *TransMes*. Similarly, major ectodermal and mesodermal populations were kept in the query dataset (fig. SS8C), including *AER-like*, *BasalEctoderm-like*, *Mesodermal-like*, *Mes_Hoxb*, *Mes_Hox13*, *Mes_Sox9*, and *Mes_Col2a1*.

The reference dataset was first preprocessed using Scanpy (*61*), which includes highly variable gene detection, data feature scaling, and PCA as previously described (*22*). Symphony (*62*) was used to correct the batch effect between sample identities (symphonypy.pp.harmony_integrate). Neighbors were calculated with the harmony-corrected PCA with the following parameters: use_rep = “X_pca_harmony,” n_neighbors = 90, n_pcs = 100. Then, the harmony-corrected PCA was mapped between the reference and the query (symphonypy.tl.map_embedding). Cells in the query dataset were projected onto the reference embedding (symphonypy.tl.ingest).

### Single-cell gene set enrichment analysis of cell fate decision genes

The potential differentiation direction of cells was determined with the AddModuleScore function in Seurat (Fig. 3F) using gene lists from the published literature (data S1) (*26*). Briefly, the average expression levels of each gene list were calculated and subtracted by the aggregated expression of control genes that were randomly selected with similar expression levels. To determine the significance between genes/modules at different time points, the Wilcoxon rank-sum test was performed.

### Sample preparation for HybISS

For in vivo limbs, pregnant female CD1 mice were purchased from Charles Rivers Laboratories, and E12.5 embryos were collected by dissecting the uterine horn. Embryos were then placed in cold 1× PBS, and the hindlimbs were isolated using fine forceps and scissors. Immediately after, the limb tissues were placed in optimal cutting temperature compound and flash-frozen in a bath with isopentane and dry ice. Tissues were then stored at −80°C until sectioning. For cryosectioning, tissues were cut at 10 μm using a cryostat (Leica CM1950) and stored at −80°C until HybISS processing. For recombinant budoids, the same protocol was performed instead of hindlimb samples.

### Hybridization-based in situ sequencing (HybISS)

A total of 131 genes were selected for HybISS to reflect the cell identity and critical pathways on the basis of the literature (data S2) (*26*). Padlock probes (PLPs) were designed using the pipeline https://github.com/Moldia/multi_padlock_design with default parameters. After target sequences were obtained, five targets were selected randomly per gene. If less than five targets were found, all the targets were selected. The backbone of the PLPs includes a 20-nucleotide ID sequence that is specific to each gene and a 20-nucleotide “anchor” that is the same for all PLPs and served only as linker sequences in this study. The designed probes were ordered from Integrated DNA Technologies, and the full list can be found in data S2.

For the step-by-step experimental protocol and details on reagents and concentrations, please refer to (*33*). Briefly, on the first day, sections were allowed to acclimate to RT, fixed with 3% paraformaldehyde, permeabilized with 0.1 M HCl, dehydrated with ethanol, and incubated in reverse transcription master mix overnight at 37°C. Following reversed transcription, the sections were postfixed with 3% paraformaldehyde and incubated in PLP mix for 30 min at 37°C and 90 min at 45°C. Then, sections were incubated in a rolling circle amplification mix for 4 hours at 37°C and overnight at 30°C. On the third day, sections were washed with 2× SSC and incubated in bridge probe (BP) mix for 1 hour at RT with light shaking. Following BP incubations, sections were washed with 2× SSC and incubated with detection oligo (DO) mix (with Hoechst) for 1 hour at RT with light shaking. Sections were then washed with PBS, and a coverslip was applied using SlowFade gold antifade mounting medium. Samples were then imaged using a Leica DMi8 epifluorescence microscope equipped with a LED light source (Lumencor SPECTRA X, nIR, 90-10172), scientific complementary metal-oxide semiconductor camera (Leica DFC9000 GTC, 11547007), and 20× objective (HC PC APO; numerical aperture, 0.8; air). After imaging, sections were incubated in a stripping buffer for 30 min to strip off DO and BP. This cycle of imaging, stripping, and incubation was repeated four more times using different BP oligos.

### HybISS image preprocessing

Each field of view was projected to obtain a flattened 2D image using a variant of the extended-depth-of-field algorithm (*63*). Each field of view was then stitched using ASHLAR (*64*), and each hybridization cycle image was aligned to the first cycle via wsireg (*65*). HybISS signals (corresponding to diffraction-limited spots) were detected with Spotiflow (*66*) by applying the pretrained HybISS model independently in each cycle and channel. Detections were assigned a gene identity (decoded) using the nearest-neighbor decoder implemented in starfish (*67*) using Spotiflow’s output probabilities as well as the spot intensities. Nuclei were segmented from the DAPI image of the first cycle with StarDist (*68*) using the 2D fluorescence versatile model. Nucleus masks were expanded for up to 20 μm each to retrieve cell masks, from which a cell-by-gene matrix was generated. Samples with detected genes lower than 180 were excluded from further analysis. Gene expressions were normalized and logarithmized using Scanpy (*61*). Differentially expressed genes across conditions were calculated using scanpy.tl.rank_genes_groups (Fig. 5B, fig. S12, and data S3).

### Morphological midline identification

The morphological midline of each budoid was identified using a custom Python tool (https://github.com/AztekinLab/MidlineIdentifier). The first step was to obtain a segmentation of the budoid structure. The binarized cell segmentation image of each budoid was subjected to image closing, hole filling, and then opening. A disc footprint with a radius of 50 was used for closing and that with a radius of 20 for opening. For budoids located less than 50 pixels from the image boundary, an additional 100 pixels were padded before segmentation. All segments were identified using skimage.measure.label and skimage.measure.regionprops, but only the largest segment was kept for structural segmentation. The remaining regions were considered as noise and were removed by setting the corresponding pixels to false. Next, the Euclidean distance transform matrix was computed on the segmentation using the scipy.ndimage.distance_transform_edt function, on which ridge detection was performed using skimage.filters.meijering with black_ridges set to false. To determine the two ends of the midline, the corners of the segmentation were detected using skimage.feature.corner_harris. The two corners closest to the two ends of the major axis based on Euclidean distance were defined as the ends of the midline to ensure that the morphological midline followed the direction of the major axis of the segmentation. Last, using the difference between one and the ridge matrix as a scoring system, the midline was calculated by walking through the two corners using skimage.graph.route_through_array. Diagonal moves were allowed.

### Cell projection

Cells that fall out of the structure segmentation were removed. Simply projecting cells onto the nearest coordinate on the morphological midline leads to an overcrowdness problem where most cells are associated with the same coordinate. Thus, we developed a distance scheme that takes into account the Euclidian distance between coordinates and cells and the number of cells associated with the coordinates. The new distance of the coordinate-cell pair is defined as

$$D_{!,\#}^{\prime }=D_{!,\#}e^{\${\%}_{!}}$$

where *D*_!,#_ represents the Euclidian distance between coordinate *i* and cell *c*. *N*_!_ is the number of cells associated with *i*. The scaling factor α is set to 0.01. Each cell was then projected to the coordinate with the shortest distance.

### Morphological midline orientation and gene quantification

To perform cross-sample comparisons, all the budoids need to be ordered in the same orientation. For this, we aimed to determine the proximal and distal domains of the morphological midline in each budoid. The two domains were determined using a set of chondrogenic (*Sox9*, *Acan*, *Col2a1*, *Col9a1*, *Col9a2*, and *Col11a1*) and fibroblast genes (*Col1a1* and *Col3a1*). Budoids were first divided into four equidistant bins along the midline, within which gene expressions were averaged following scanpy.tl.score_genes to calculate the enrichment score of the proximal and distal genes, respectively. The half with a higher chondrogenic score was determined as the proximal. Midlines were then scaled to the range of zero to one to make it comparable across different budoids, where zero represents the proximal, and one represents the distal. For line plots in fig. S14B, *statsmodels.ZeroInflatedPoisson* was used to fit the raw expression of each condition.

The proximal and distal scores were further used to classify the polarization property of budoids. Budoids with both proximal and distal scores greater than 0.01 were considered as polarized, budoids with either a proximal score or distal score less than 0.01 were considered as nonpolarized, and budoids with both proximal and distal scores less than 0.01 were considered invalid samples. Polarized budoids can be divided into two equal parts from the middle along the morphological midline, the proximal and the distal.

### Principal components analysis (PCA)

Gene expression of each budoid or each part (proximal or distal) of the budoid was averaged. Genes expressed in less than 1% of cells were excluded. Missing values were imputed using the means. The averaged expression matrix was standardized using sklearn.preprocessing.StandardScaler, followed by PCA using sklearn.decomposition.PCA (Fig. 5C).

### Identification of the most representative section

For different sections from the same budoid, we remove the outlier sections on the basis of the PCA. If these sections show different phenotypes (e.g., one polarizes but not the other), we remove all the sections. If these sections are similar, we (i) keep the middle section; (ii) if there are sections from other budoids in the same slice that have already been retained, keep the section of similar morphological positions as the retained ones; and (iii) keep the morphologically more intact ones.

### Statistics and replicate information

Sample sizes were not predetermined, and blinding was not applied during analyses. A one-way analysis of variance (ANOVA) was used in Fig. 1 (E and L) and figs. S9 (D and E) and S11 (A and B), and a *t* test was used for Fig. 4G and fig. S10B. Other performed statistical tests were indicated in the methods in relevant sections. Replicate information for experiments is provided in the text or legends. Throughout the manuscript, *n* denotes technical replicates, and *N* denotes a biological replicate that is performed on a separate day, with a separate material, or both. All representative images are selected from experiments with at least *n* = 3 and *N* = 3. The standard error of the mean was provided for relevant data with (±standard error of the mean).
