# Supplementary material for: Specialized signaling centers direct cell fate and spatial organization in a mesodermal organoid model
Source: Sci Adv. 2025 Nov 28;11(48):eady7682. doi: 10.1126/sciadv.ady7682 (PMC12662206; doi:10.1126/sciadv.ady7682)
Supplement: Supplementary file 1 — Figs. S1 to S14 Legends for data S1 to S4 [file sciadv.ady7682_sm.pdf]

Science Advances
AAAS

Supplementary Materials for

Specialized signaling centers direct cell fate and spatial organization in a mesodermal organoid model

Evangelia Skoufa *et al.*

Corresponding author: Can Aztekin, can.aztekin@tuebingen.mpg.de

Sci. Adv. **11**, eady7682 (2025)
DOI: 10.1126/sciadv.ady7682

The PDF file includes:

Figs. S1 to S14
Legends for data S1 to S4

Other Supplementary Material for this manuscript includes the following:

Data S1 to S4

Fig. S1.

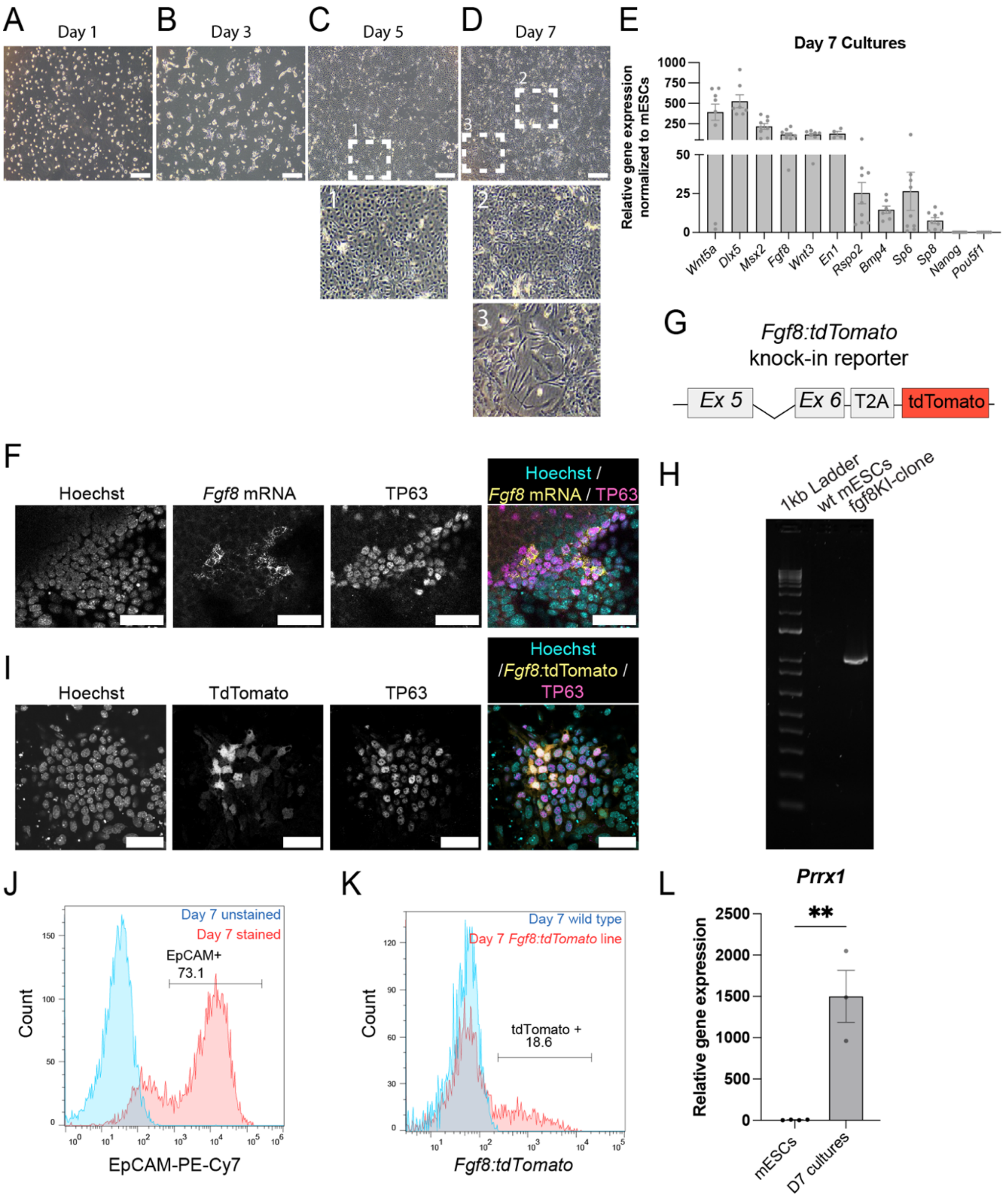

Supplementary Fig.1: **Characterization of stem-cell-derived heterogeneous cultures.**

(A-D) Representative brightfield images of induced cultures on Day 1 (A), Day 3 (B), Day 5 (C), and Day 7 (D). Insets highlight the homogeneous induction observed on Day 5 and the mixture of epithelial and mesenchymal cells at Day 7. Scale bar = 50 µm.

(E) Quantitative RT-PCR results of the apical ectodermal ridge (AER) and pluripotent stem cell markers for Day 7 cultures. All samples were normalized to mouse embryonic stem cells (mESCs) and RPL27 housekeeping gene expression. Each dot represents the average of at least two technical replicates in a biological replicate. N>7 for all analyzed genes.

(F) Representative confocal image of a cluster of *Fgf8* mRNA and TP63-positive AER-like cells. Cyan: Hoechst, Yellow: Fgf8 mRNA, magenta: TP63. Scale bar = 50 µm.

(G) Schematic describing the knock-in to generate *the Fgf8:tdTomato* reporter line.

(H) Genotyping result for the generated *Fgf8* reporter line, with primers targeting Exon 6 and *tdTomato*.

(I) Representative confocal image of a cluster of *Fgf8:tdTomato* and TP63-positive AER-like cells. Cyan: Hoechst, Yellow: *Fgf8:tdTomato*, magenta: TP63. Scale bar = 50 m.

(J) An example of flow cytometry data used to quantify EpCAM-positive cells in Day 7 cultures, leading to the Fig. 1E, is shown. Unstained samples were used to distinguish the signal.

(K) An example of flow cytometry data to quantify Fgf8:tdTomato positive cells in Day 7 cultures, leading to the Fig. 1F, is shown. Wild-type controls were used to distinguish the signal.

(L) Quantitative RT-PCR results of *Prrx1* expression in Day 7 (D7) cultures. All samples are normalized to RPL27 housekeeping gene expression. Each dot represents the average of at least two technical replicates in a biological replicate. N=4 for mESCs, and N=3 for Day 7 cultures.

Fig. S2.

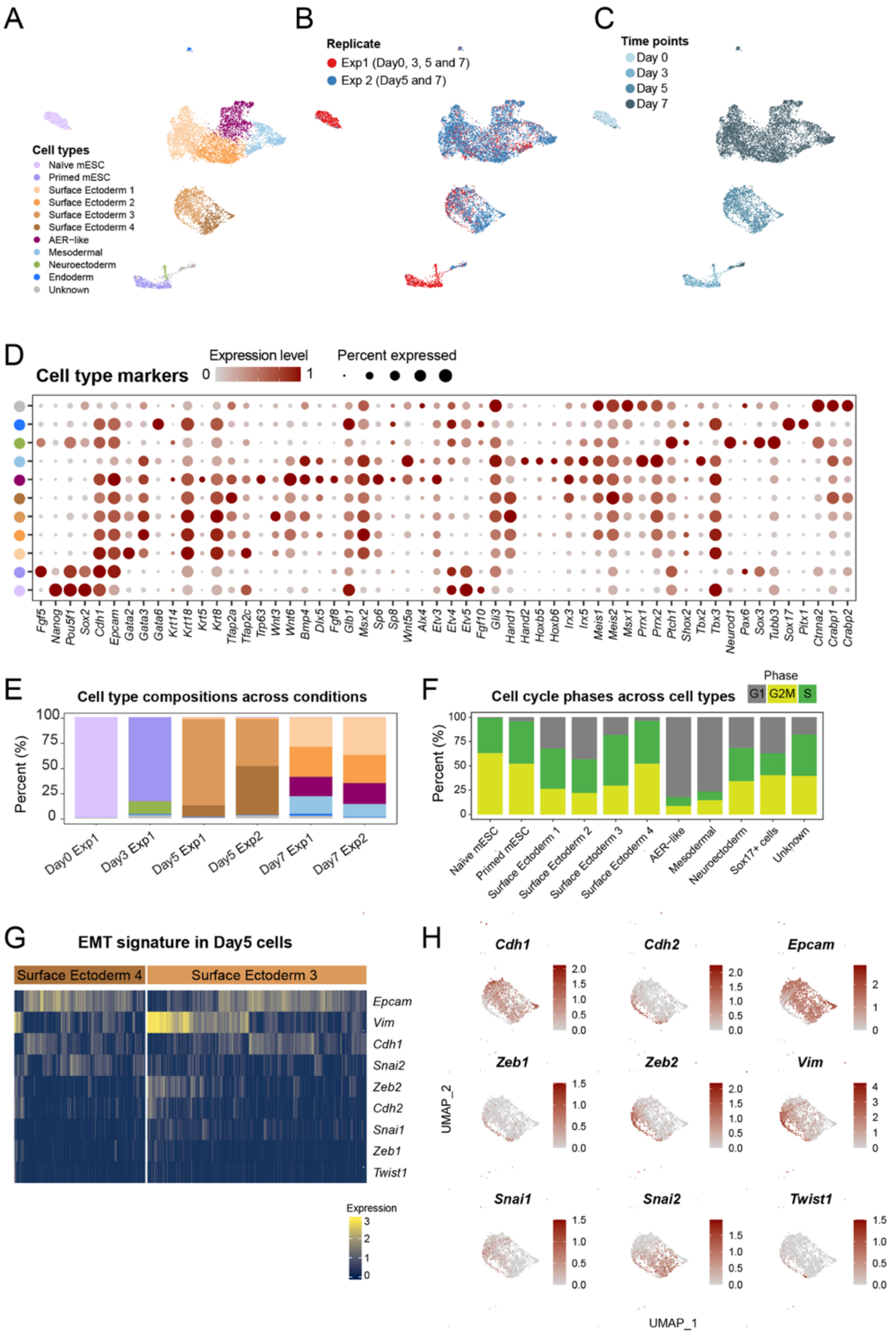

Supplementary Fig.2: Single-cell RNA sequencing-based characterization of heterogeneous cultures.

(A) UMAP representation of single-cell RNA sequencing (scRNA-Seq) displaying processed samples across different time points of the induction protocol.

(B) UMAP representation of replicates. Replicate 1 contains cells from day 0, 3, 5, and 7 of the protocol, represented by red cells. Replicate 2 contains cells from day 5 and 7 of the protocol, represented by blue cells.

(C) UMAP representation of cells from different time points in the dataset. Shades of light to dark blue indicate day 0 to day 7 samples.

(D) Dot plot showing marker expressions used to annotate cell types. The size of the dot represents the percentage of cells expressing each marker.

(E) Proportion analysis showing the relative abundance of each annotated cell type within the culture at each time point and replicate. Please note that some of these data are the same as in Fig 1H.

(F) ScRNA-Seq-based cell cycle analysis for detected clusters.

(G) Heatmap showing epithelial-to-mesenchymal transition (EMT) associated gene expression in day 5 surface ectoderm clusters. Each column represents one cell.

(H) UMAP showing EMT-associated gene expression in day 5 surface ectoderm clusters. Shades of red indicate the expression level.

Fig. S3.

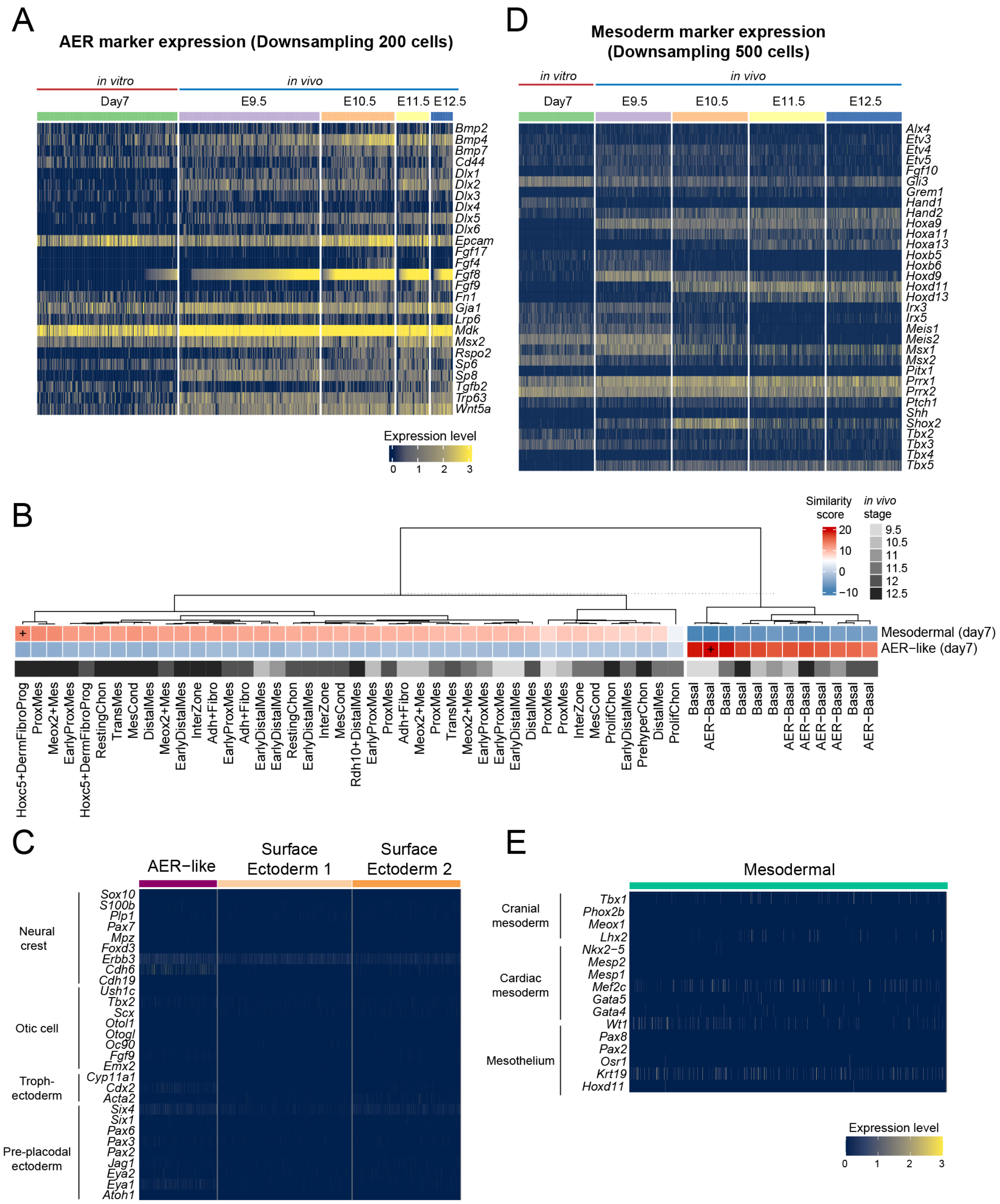

Supplementary Fig.3: Heterogeneous cultures show varying degrees of similarity to in vivo limb cells.

(A) Heatmap showing AER-associated gene expression levels in the generated *in vitro* AER-like cluster and *in vivo* AER clusters at developmental stages E9.5 to E12.5. *In vivo* data are obtained

from Allou et al. (E9.5) (*64*) and Desanlis et al. (E10.5-12.5) (*65*). Each column represents one cell.

(B) Heatmap displaying the transcriptome-wide similarity of generated in vitro AER-like and mesodermal cells at day 7 with in vivo limb cell types across stages E9.5 to E12.5. A '+' symbol denotes the highest level of similarity of each row, and varying shades of gray at the bottom of the heatmap indicate the *in vivo* developmental stages. *In vivo* data and cluster annotations are obtained from Zhang et al.(*22*).

(C) Heatmap illustrating the expression levels of relevant ectodermal cell type markers in the generated *in vitro* AER-like cluster and surface ectoderm clusters in day 7 cultures. Each column represents one cell.

(D) Heatmap presenting the expression levels of early lateral plate mesoderm and limb bud mesoderm markers in the generated in vitro mesoderm cluster compared with in vivo limb bud mesoderm clusters at stages E9.5 to E12.5. *In vivo* data and cluster annotations are obtained from Allou et al. (E9.5) (*64*) and Desanlis et al. (E10.5-12.5) (*65*) . Each column represents one cell.

(E) Heatmap showing relevant mesodermal cell type marker expression levels in the generated *in vitro* mesodermal cluster at day 7 cultures.

Fig. S4.

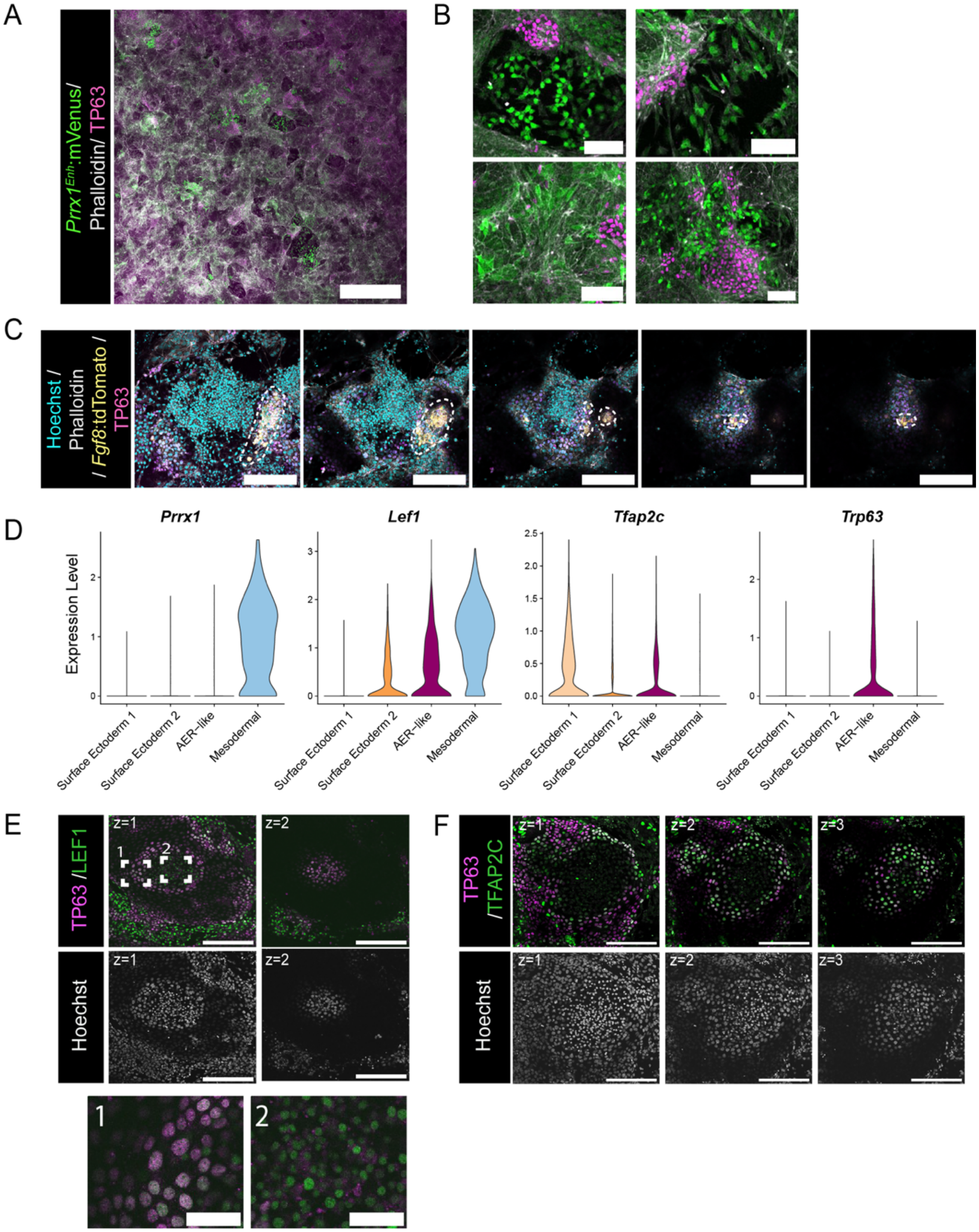

Supplementary Fig.4: Generated epithelial and mesenchymal cells self-organize into domes.

(A) Representative confocal image showing the distribution of *Prrx1^{Enh}:mVenus* and TP63 positive cells in day 7 cultures. Green: *Prrx1^{Enh}:mVenus*, Gray: Phalloidin, Magenta: TP63; Scale bar = 1000 μm.

(B) Higher magnification images of *Prrx1^{Enh}:mVenus* and TP63-positive cells within day 7 cultures. Note the fibroblast-like and circular morphologies of the *Prrx1^{Enh}:mVenus* positive cells. Green: *Prrx1^{Enh}:mVenus*; White: Phalloidin; Magenta: TP63. Scale bar = 100 µm.

(C) Sequential Z-stack confocal images of a dome. The left to right panels show progressing depth through the Z-stack. Note that *Fgf8:tdTomato*-positive cells are located at the corners and tip of the dome and are circled with a dashed line. Z-stacks are denoted by z numbers. Cyan: Hoechst; White: Phalloidin; Yellow: *Fgf8:tdTomato*; Magenta: TP63. Scale bar = 200 µm.

(D) Violin plots illustrating *Prrx1*, *Lef1*, *Tfap2c*, and *Tp63* expression levels across the main identified clusters in the day 7 scRNA-Seq dataset. Note that *Lef1* positive/*Tfap2c* negative/*Trp63* negative cells only indicate the mesodermal cluster.

(E) Sequential Z-stack confocal images of a dome. Left to right panels represent progressing depth through the Z-stack, denoted by z numbers. Note that the larger nuclei of TP63-positive cells form the outer layer compared to LEF1-positive cells at the core of the dome. Gray: Hoechst; Green: LEF1; Magenta: TP63. Scale bar = 200 µm. Insets 1 and 2 offer higher magnification views of selected areas. Scale = 50 µm.

(F) Sequential Z-stack confocal images of a dome structure. Left to right panels represent progressing depth through the Z-stack, denoted by z numbers. Green: TFAP2C; Magenta: TP63; Gray: Hoechst. Scale bar = 200 µm.

Fig. S5.

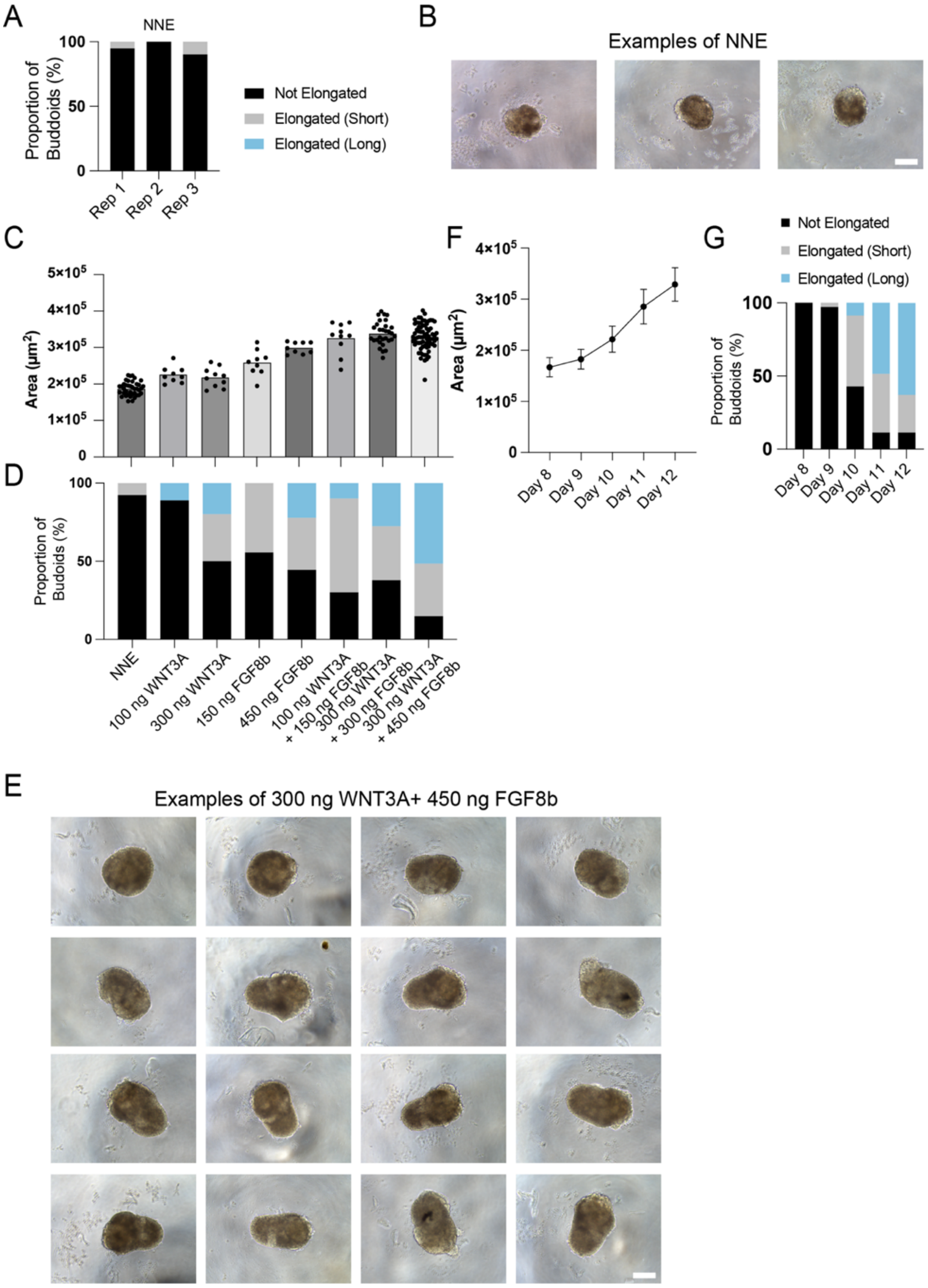

Supplementary Fig.5: FGF8b and WNT3a treatment enhance the efficiency of budoid formation from heterogenous cultures-derived aggregates.

(A) Bar chart illustrating the proportion of budoids elongating in no treatment condition at day 12. The total number of aggregates analyzed was n = 39 and N = 3.

(B) Brightfield images of no treatment condition on day 12. Scale bar = 100 µm.

(C-D) (C)Bar plot demonstrating the day 12 budoids area resulting from various FGF8b and WNT3a treatment concentrations. (D) Bar chart displaying the proportion of budoids exhibiting no elongation, elongation (short), or elongation (long) after treatment with different concentrations of growth factors. NNE is a treatment condition. NNE: n= 39, N=3: 100 ng/ml WNT3A: n= 9, N=1: 300 ng/ml WNT3A n=10, N=1; 150 ng/ml FGF8b, n=9, N=1: 450 ng/ml FGF8b n=9, N=1: 100 ng/ml WNT3A and 150 ng/ml FGF8b, n=10, N=1: 300 ng/ml WNT3A and 300 ng/ml FGF8b, n=30, N=2; 300 ng/ml WNT3A and 450 ng/ml FGF8b, n=72, N=2. Please note that some of these data are the same as in Fig 3B.

(E) Examples of brightfield images showing day 12 budoids with varying elongation phenotypes after treatment with 300 ng WNT3A and 450 ng FGF8b. Scale bar = 100 µm.

(F-G) (F) Line plot depicting the change in area from day 8 to day 12 of the protocol for budoids under the influence of FGF8b and WNT3a treatment. (G) Bar chart presenting the elongation phenotypes of budoids over time. Total aggregates analyzed: n = 94 for days 8, 9, 11, and 12; and n= 45 for day 10, N=2.

Fig. S6.

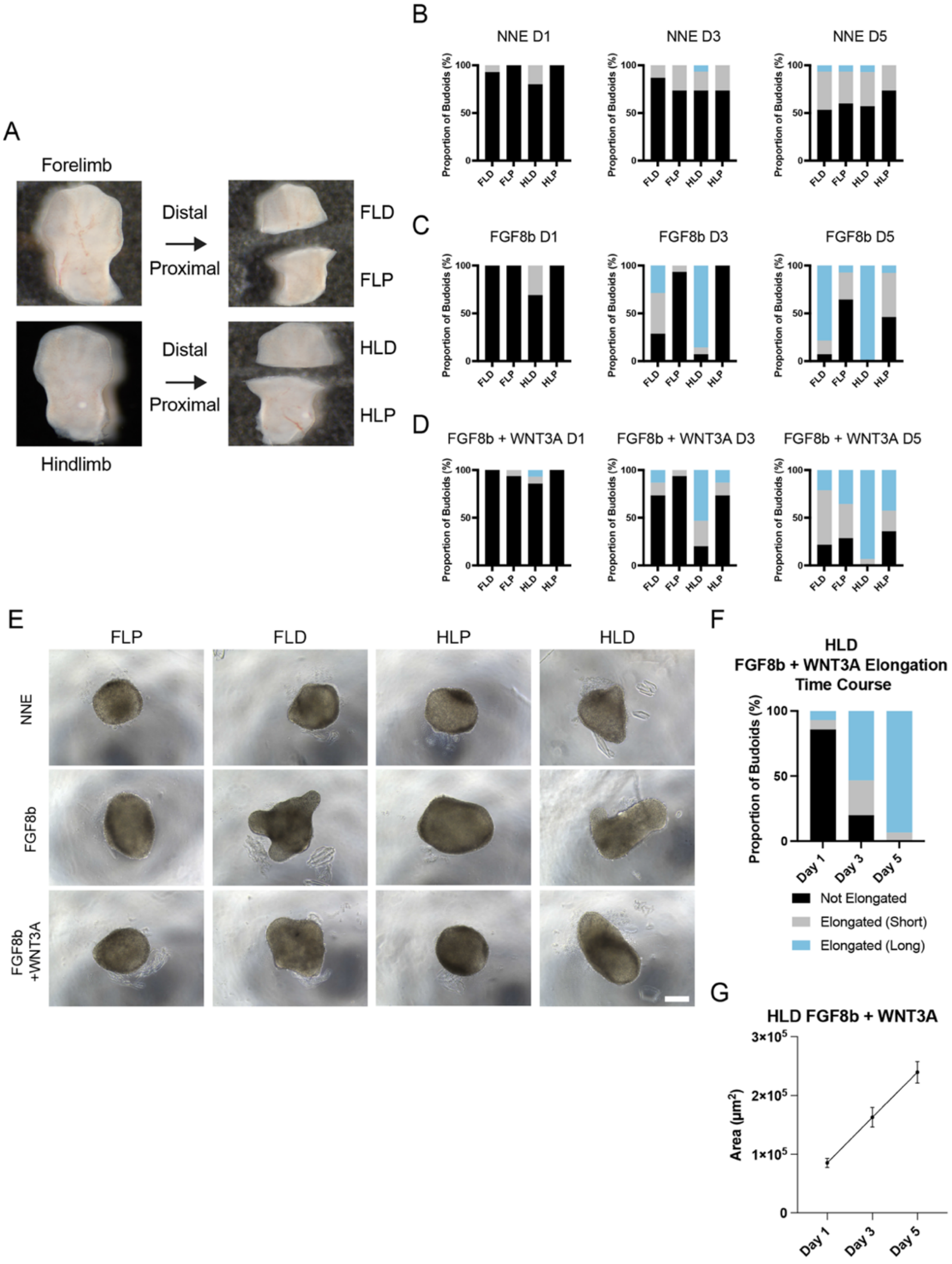

Supplementary Fig.6: FGF8b and WNT3a treatment enhance the formation efficiency of in vivo-derived budoids.

(A) Images of dissected forelimbs and hindlimbs from E12.5 mouse embryos used to generate budoids. The forelimb distal (FLD), forelimb proximal (FLP), hindlimb distal (HLD), and hindlimb proximal (HLP) regions are indicated.

(B-D) Bar charts showing the proportion of budoids exhibiting various elongation phenotypes on Day 1, Day 3, and Day 5 after treatments with NNE, FGF8b, and a combination of FGF8b with WNT3a. The total number of budoids analyzed was n >14 for all samples, N = 3.

(E) Brightfield images of Day 5 budoids displaying varying elongation phenotypes after treatments with NNE, FGF8b, and a combination of FGF8b with WNT3a across different collected limb samples. Scale bar = 100 µm.

(F) Bar chart illustrating the time-course elongation phenotypes of HLD budoids treated with FGF8b and WNT3a. The total number of budoids analyzed n = 15, N = 3.

(G) Line plot showing the time course area of HLD budoids treated with FGF8b and WNT3a. The total number of budoids analyzed n =15, N = 3.

Fig. S7.

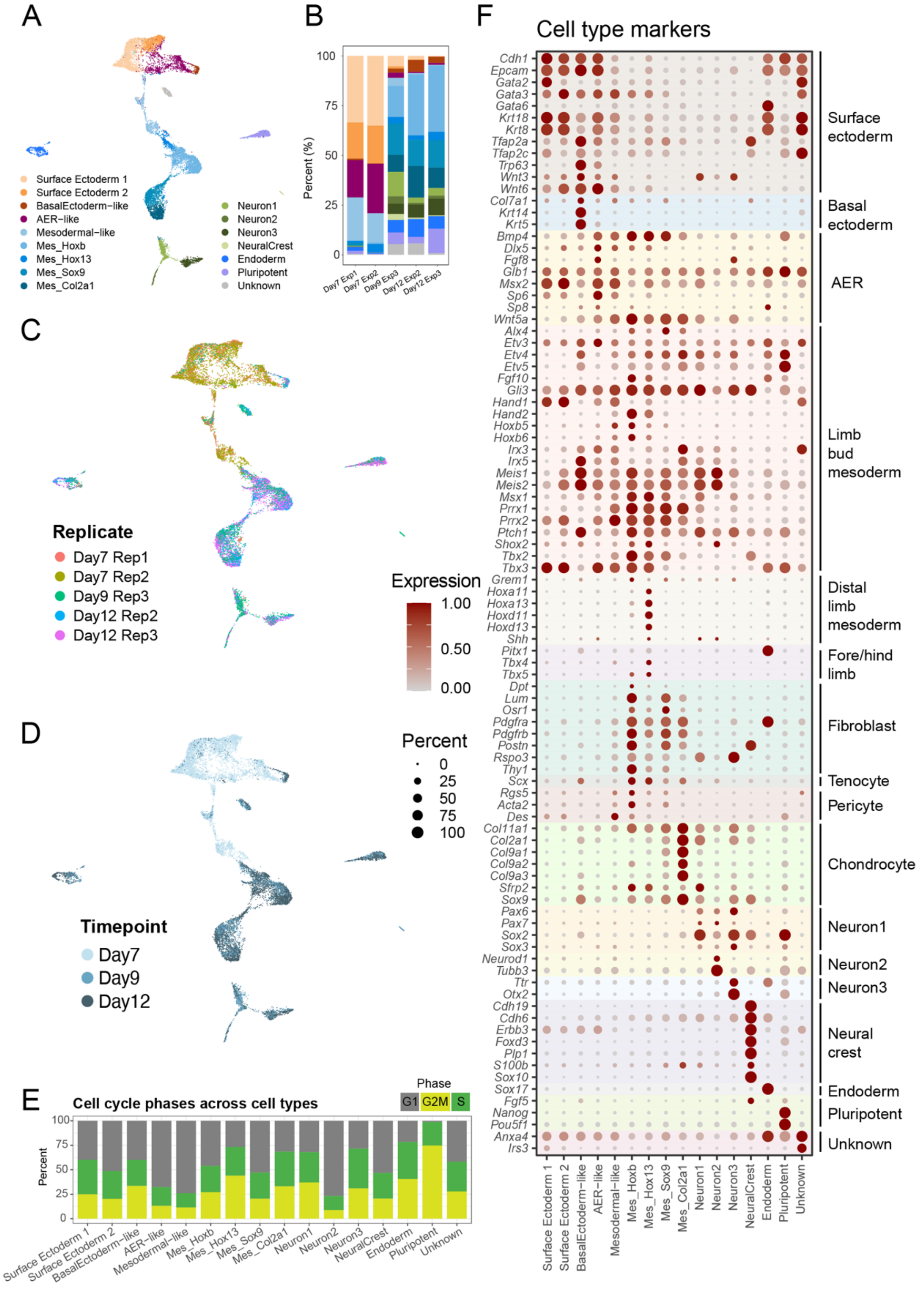

Supplementary Fig.7. Single-cell RNA sequencing-based characterization of budoids

(A) UMAP visualization of scRNA-Seq data from budoids at different culture time points, as highlighted in Fig 3A, with each cell type uniquely colored.

(B) Bar chart representation of the relative proportions of the cell types identified in the scRNA-Seq analysis.

(C) UMAP plot showing the distribution of cells from different replicates.

(D) Temporal UMAP representation of cells color-coded by time point from day 7 (light blue) to day 12 (dark blue) samples.

(E) Bar chart summarizing the cell cycle phase distribution across all detected cell populations.

(F) Dot plot characterizing the expression of marker genes that define each cell type. Dot size corresponds to the percentage of cells expressing a particular gene, while color intensity reflects expression level. Please note that the genes annotated with specific cell identities are not exclusive and can be expressed across different cell types.

Fig. S8.

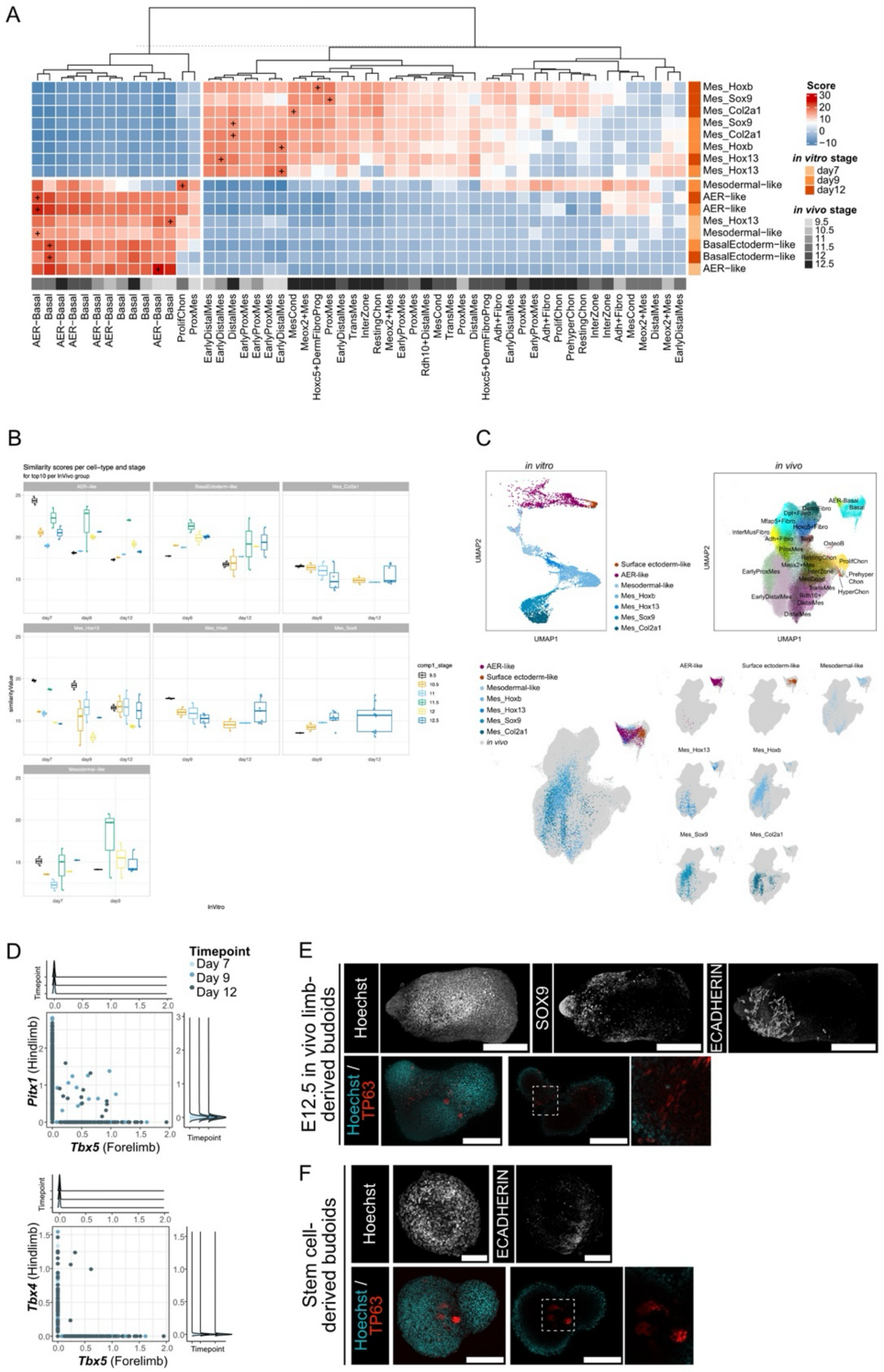

Supplementary Fig.8: Stem cell-derived budoids show similarities to developing limbs *in vivo*, and ectodermal cells are internalized in stem cell or *in vivo*-derived budoids.

(A) Heatmap presenting the transcriptome-level similarities between *in vitro*-generated AER-like and mesodermal cells during budoids generation over days 7 to 12, and *in vivo* limb cell types at

developmental stages E9.5 to E12.5. '+' indicates the highest similarity in each row, while the gray gradient at the bottom of the heatmap reflects the developmental stages. *In vivo* data and cluster annotations are obtained from Zhang et al. (*22*).

(B) The similarity values of the ten most similar in vivo populations compared to the *in vivo* limb cells, for each budoid cluster.

(C) (Top-Left) The UMAP shows the major cell types used in the analysis, which represents a subset of data presented in Fig. 3B. (Top-Right) The reference UMAP of the mouse limb buds. Cells are coloured by clusters. which represents a subset of data adapted from Zhang et al. (*22*). (Bottom) Reference mapping of major cell types generated in 3D cultures (Fig. 3B) in an aggregated view (left) and split view (right). Cells are colored by cell types.

(D) Scatter plots demonstrating expression patterns of hind and fore limb identity-related genes in individual cells across various time points. Each dot represents a single cell, and time points are distinguished by color shading.

(E) (Top) Max projection confocal image showing day 5 in vivo-derived budoids with polarized SOX9 expression and limited E-CADHERIN. Scale bar = 200 µm. (Bottom-left) Max projection confocal image showing TP63-positive cells. (Bottom-mid) An optical section of the budoid shows TP63-positive cells internally localized within the structure. Scale bar = 200 µm. (Bottom-right) Zoomed-in view of the optical section.

(F) (Top) Max projection confocal image showing day 9 in stem cell-derived budoids with limited E-CADHERIN. Scale bar = 200 µm. (Bottom-left) Max projection confocal image showing day 12 budoid with TP63 positive cells. (Bottom-mid) An optical section of the budoid shows TP63-positive cells internally localized within the structure. Scale bar = 200 µm. (Bottom-right) Zoomed-in view of the optical section.

Fig. S9.

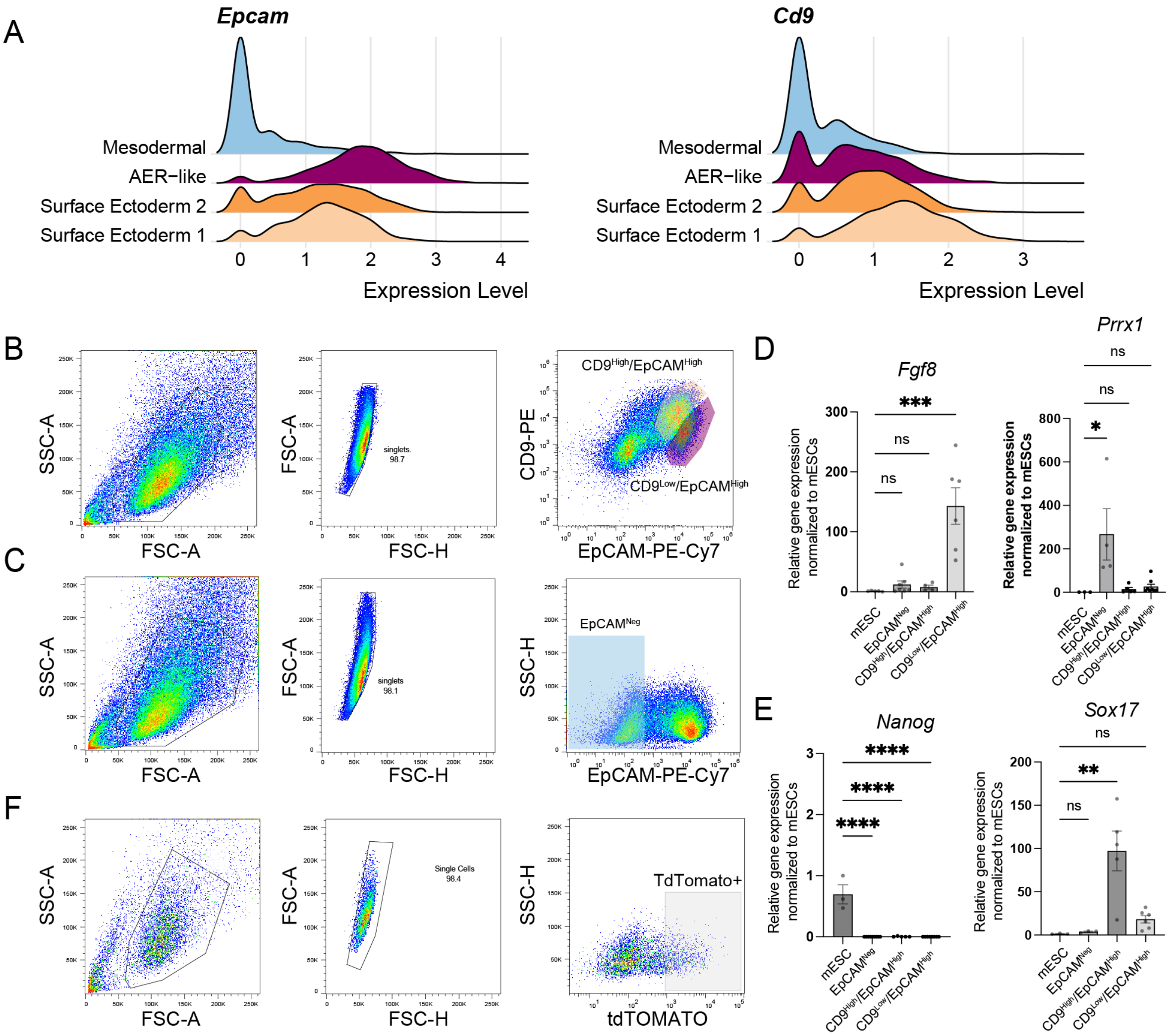

Supplementary Fig.9. Cell sorting strategy to enrich mesodermal, AER-like, and surface-ectoderm-like cells

(A) Histogram plots illustrate the expression of *Epcam* and *Cd9* in Day 7 clusters identified by scRNA-seq data.

(B) Representative flow cytometry data showing antibody-based labeling with CD9 and EpCAM generates three populations. An enrichment strategy based on this is outlined for AER-like ($CD9^{Low}/EPCAM^{High}$) and surface-ectoderm-like ($CD9^{High}/EPCAM^{High}$) populations. FACS-enriched populations are shown with shaded red for AER-like cells and yellow for surface ectoderm-like cells.

(C) Representative flow cytometry data showing antibody-based labeling with EpCAM for isolating mesodermal cells ($EpCAM^{Neg}$). The FACS-enriched population is shown in shaded blue.

(D) Quantitative RT-PCR results for (left) *Fgf8* and (right) *Prrx1* in the FACS-enriched populations against mouse embryonic stem cells (mESCs). All samples are normalized to RPL27 housekeeping gene expression. Each dot represents the average of at least 2 technical replicates for one biological replicate. N >3 for all conditions.

(E) Quantitative RT-PCR results for (left) *Nanog* and (right) *Sox17* in the FACS-enriched populations against mESCs. All samples are normalized to RPL27 housekeeping gene expression. Each dot represents the average of at least 2 technical replicates for one biological replicate. N >3 for all conditions.

(F) Representative flow cytometry data showing the enrichment strategy for isolating AER-like cells using the *Fgf8:tdTomato* reporter line. The FACS-enriched population is shown with shaded gray.

Fig. S10.

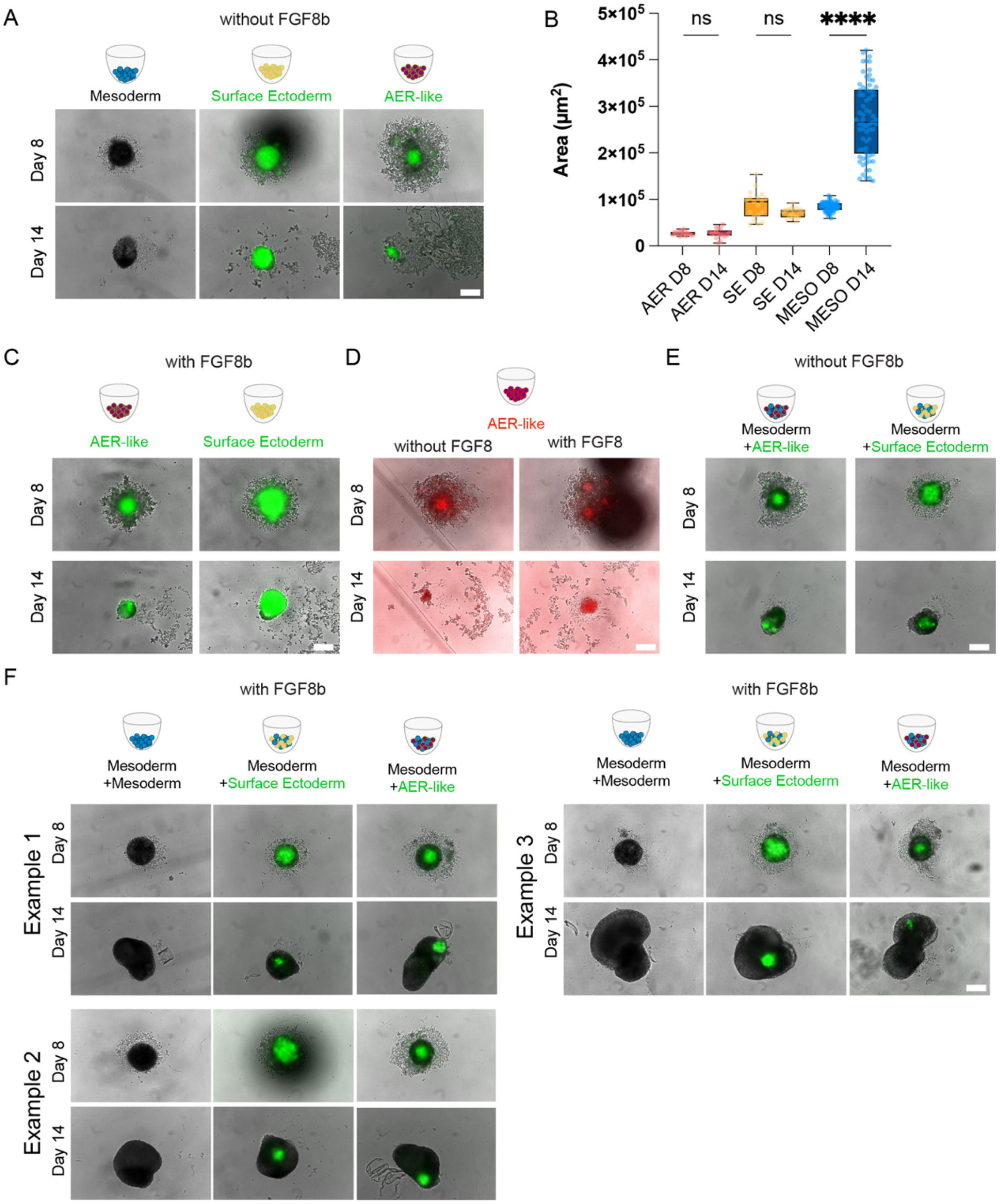

Supplementary Fig.10. FGF8b treatment is necessary for budoids generation

(A) Representative images display FACS-enriched EpCAM^{Neg} mesodermal, CD9^{High}/EpCAM^{High} surface ectoderm-like, CD9^{Low}/EpCAM^{High} AER-like populations without FGF8b treatment at Day 8 and Day 14 of the protocol. Note excessive non-aggregated and shed cells, especially in ectodermal cells. Scale bar = 100 µm.

(B) A box plot indicates the changes in area measurements for the FACS-enriched populations on Day 8 and Day 14 of the protocol. D8 denotes day 8 of the protocol, and D14 denotes day 14 of the protocol. Total number of aggregates from AER-like n= 17 from N=4 , SE-like= 19 from N=4, and mesodermal n= 69, from N= 9, n.s.=not significant; **** denotes p= <0.0001;

(C) Representative images showing FACS-enriched CD9^{Low}/EpCAM^{High} AER-like and CD9^{High}/EpCAM^{High} surface ectoderm-like cells with FGF8b treatment at Day 8 and Day 14. Please note excessive non-aggregated and shed cells, particularly in day 8 ectodermal cells. Scale bar = 100 μm.

(D) Representative images showing FACS-enriched *Fgf8:tdTomato* positive AER-like cells from *Fgf8:tdTomato* mESC line, with and without FGF8b treatment. Note excessive non-aggregated and shed cells. Scale bar = 100 μm.

(E) Representative images of recombination of mesoderm with AER-like or surface ectoderm-like cells without FGF8b treatment at Day 8 and Day 14 of the protocol are shown. Note excessive non-aggregated and shed cells, especially in day 8 ectodermal cells. Scale bar = 100 μm.

(F) Additional examples of recombinant budoids with FGF8b treatment at Day 8 and Day 14 of the protocol. These images show the range of phenotypes recorded, albeit lesser in frequency compared to those shown in Fig. 4 C-E. Images in the same example group show structures from the same experiment. Note excessive non-aggregated and shed cells, especially in ectodermal cells. Scale bar = 100 μm.

Fig. S11.

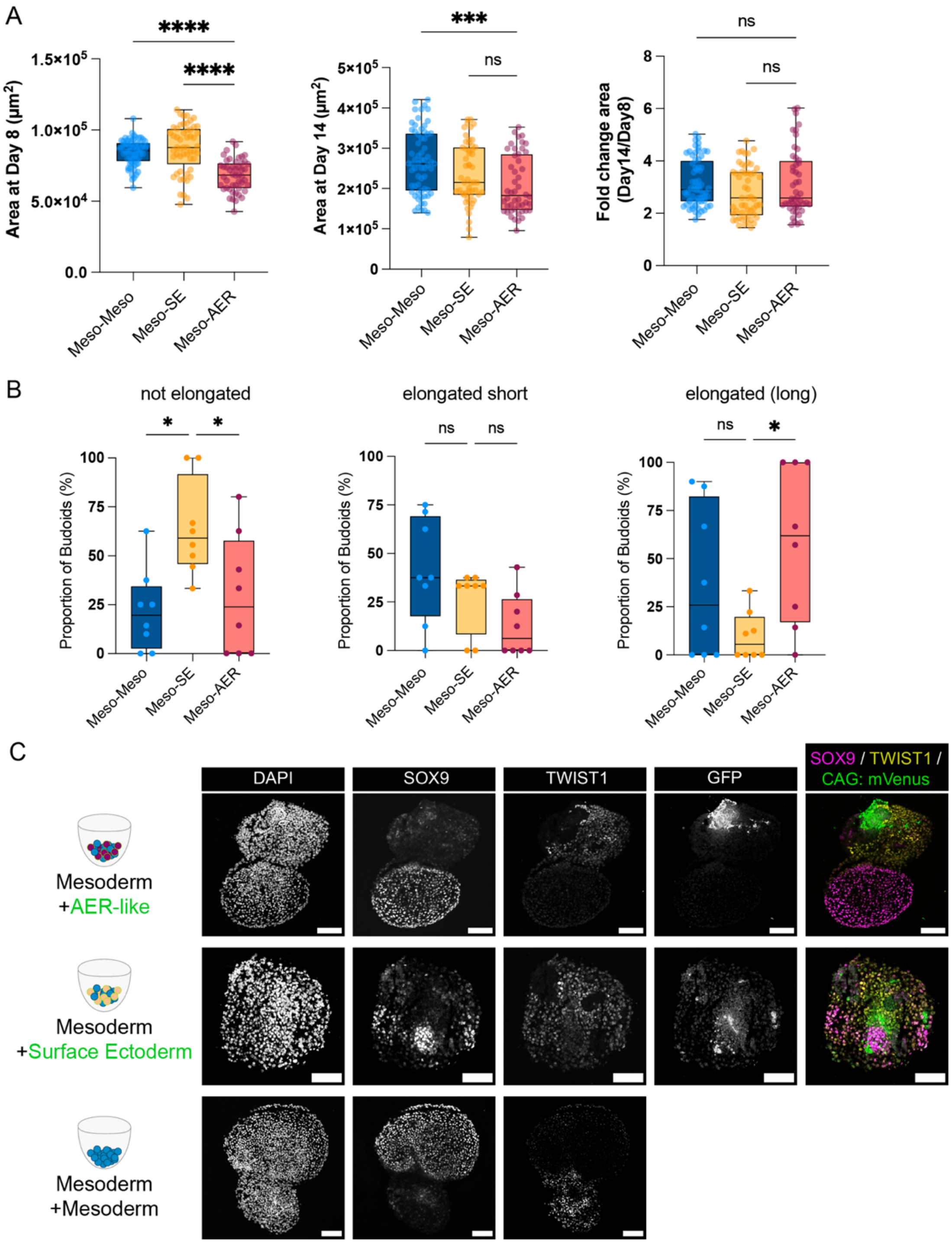

Supplementary Fig.11. Recombinations show no significant difference in area fold change, but they influence spatial organization

(A) Box plots illustrating area measurements for FACS-enriched populations on (Left) day 8, (Mid) day 14, and (Right) the fold change in area from day 8 to day 14 for individual budoids.

Each dot represents one recombinant budoid. The total number of budoids analyzed for Meso-Meso n= 64; Meso-SE n=53, Meso-AER n= 48, all from N=8. n.s.= not significant; *** denotes $p < 0.0005$, **** denotes $p < 0.0001$;

(B) Box plots depicting the proportions of budoids exhibiting various elongation phenotypes in recombination experiments for (Left) non-elongated, (Middle) elongation (short), and (Right) elongation (long). Each dot represents the proportion of one biological replicate obtained from 3-10 technical replicates. N= 9.

(C) Representative confocal images of sectioned recombinant budoids are provided to illustrate the spatial organization within these structures. Magenta: SOX9, Yellow: TWIST1, Green: GFP. Scale bar = 100 μm.

Fig. S12.

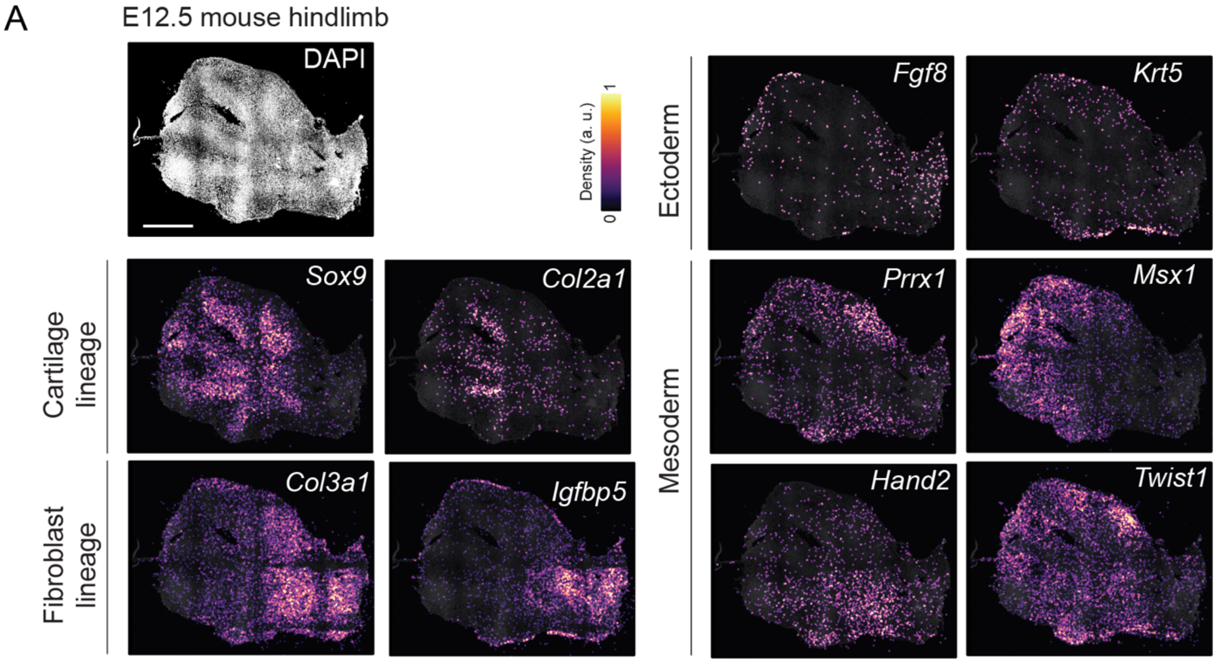

Supplementary Fig.12. **Hybridization-based in situ sequencing (HybISS) detects known limb developmental gene expression patterns in mouse hindlimb**

Example Hybridization-based in situ sequencing (HybISS) results for limb development-associated genes in *in vivo* stage E12.5 mouse hindlimb. Scale bar = 500 µm.

Fig. S13.

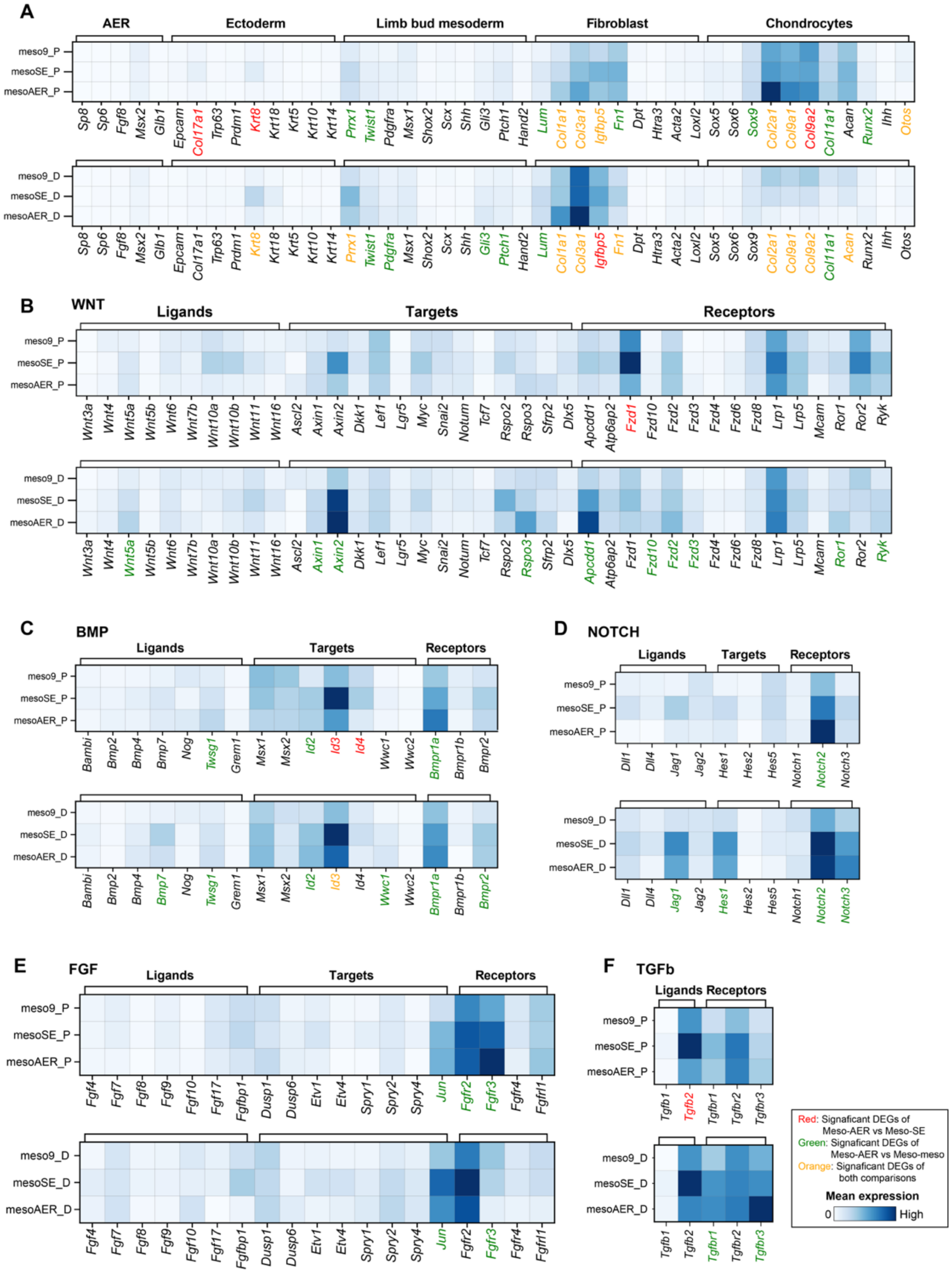

Supplementary Fig.13. HybISS reveals recombination-induced molecular changes in proximal and distal domains

Heatmaps showing average expression levels of genes associated with (A) limb development-related cell fates, (B) WNT, (C) BMP, (D) NOTCH, (E) FGF, and (F) TGFb pathways in the

recombinant budoids single cell distal and proximal domains. Genes with statistically significant differences (adjusted P-values < 0.05) are highlighted, with green indicating a significant difference between mesoderm-AER and mesoderm-mesoderm comparisons, red signifies significant differences between mesoderm-AER and mesoderm-surface ectoderm and orange indicating significance in both comparisons. Number of HybISS sections analyzed for Meso-Meso n= 20, N=4, Meso-SE n=12, N=3, Meso-AER n=20, N= 4.

Fig. S14.

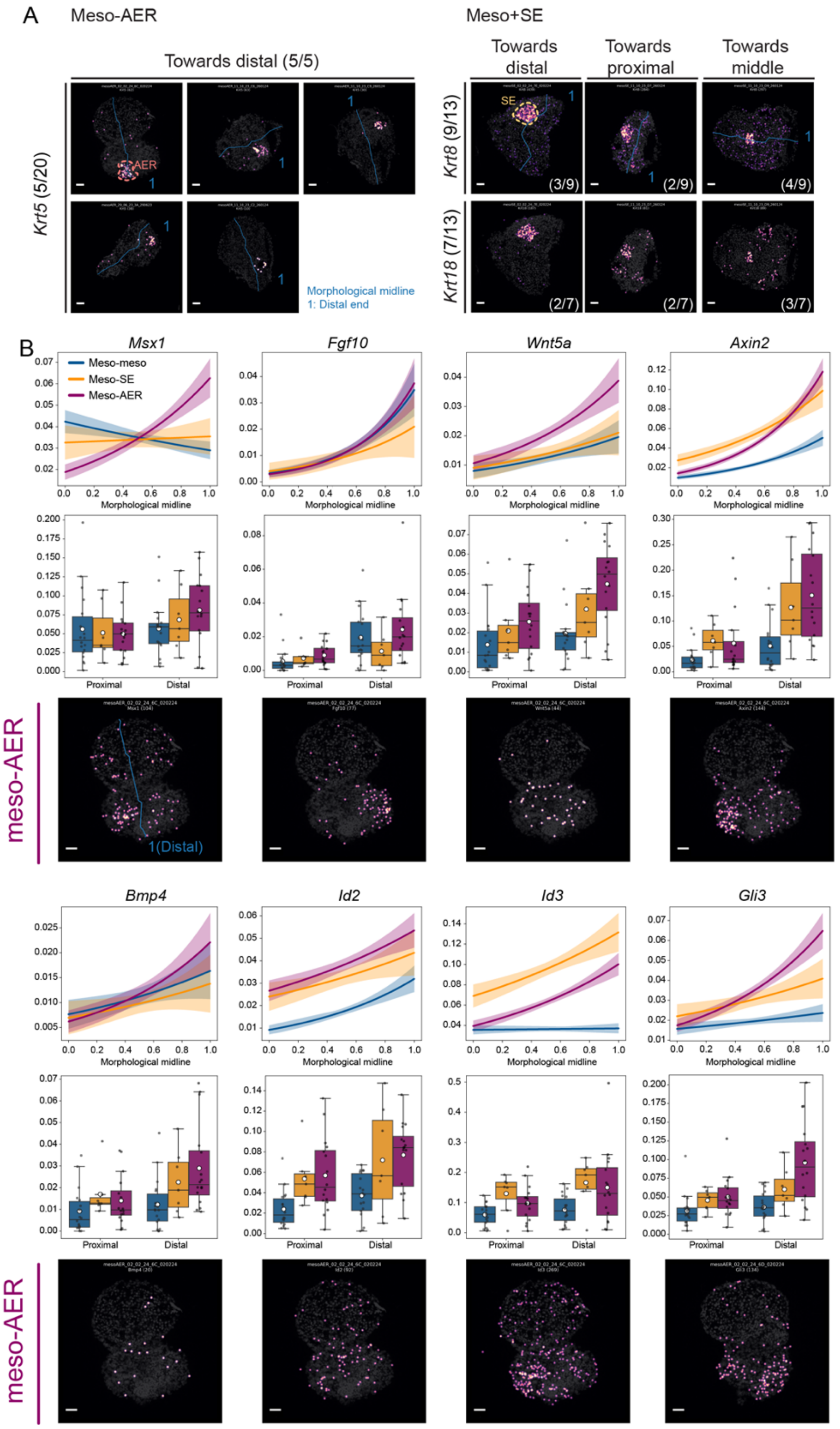

Supplementary Fig.14. HybISS demonstrates the lasting impact of AER-like recombinations

(A) (Left) *Krt5* expression marks the remnants of AER-like cells in meso-AER recombinant sections, with a 5/20 prevalence. Notably, all 5/5 cases with *Krt5* positive cells are localized to the

distal domain of the recombinants. (Right) In meso-SE recombinants, remnants of surface-ectoderm-like cells are identifiable through *Krt8* in 9/13 instances and *Krt18* in 7/13 instances. These markers do not exhibit preferential expression towards distal or proximal domains. Scale bar =50 μm.

(B) Previously suggested AER induced *Msx1, Fgf10, Wnt5a, Axin2, Bmp4, Id2, Id3*, and *Gli3* in recombinant budoids are shown. For each gene: (Top) Line graphs plot the gene expression gradients from the proximal to distal ends of recombinant budoids. Each condition is color-coded. The zero-inflated Poisson model was used to fit the data in each condition. Confident intervals were shown in shades. (Middle) Box plots show the normalized expression levels of these genes within distal versus proximal domains across different conditions. (Bottom) Accompanying example images display HybISS results for the specified genes in meso-AER samples. Scale bar =50 μm.

Data S1.

Quality Control Single-cell RNA-sequencing analysis

Data S2.

HybISS PLP synthesis sequences

Data S3.

HybISS DEGs list

Data S4.

Primer sequences
